# Attitude Control of Ornithopter Wing by Using a MIMO Active Disturbance Rejection Strategy

**DOI:** 10.3390/s23146602

**Published:** 2023-07-22

**Authors:** Josiel Alves Gouvêa, Luciano Santos Constantin Raptopoulos, Milena Faria Pinto, Elkin Yesid Veslin Díaz, Max Suell Dutra, Lucas Costa de Sousa, Victor Manuel Oliveira Batista, Alessandro Rosa Lopes Zachi

**Affiliations:** 1 Department of Control Systems and Automation Engineering, Federal Center of Technological Education of Rio de Janeiro, Nova Iguaçu 26.041-271, Brazil; josiel.gouvea@cefet-rj.br (J.A.G.); luciano.raptopoulos@cefet-rj.br (L.S.C.R.); 2 Graduate Program in Electrical Engineering, Federal Center of Technological Education of Rio de Janeiro, Rio de Janeiro 20.271-110, Brazil; milena.pinto@cefet-rj.br (M.F.P.); lucas.sousa.1@aluno.cefet-rj.br (L.C.d.S.); victor.manuel@aluno.cefet-rj.br (V.M.O.B.); 3 Electromechanical Engineering Program, Santander Technology Units, Bucaramanga 680006, Colombia; eyveslin@correo.uts.edu.co; 4 Mechanical Engineering Program, Federal University of Rio de Janeiro, Rio de Janeiro 21.941-598, Brazil; max@mecanica.coppe.ufrj.br

**Keywords:** MIMO uncertain systems, modified-plant ADRC, disturbance rejection, attitude control, ornithopter wing control

## Abstract

This work proposes a mathematical solution for the attitude control problem of an ornithopter wing. An ornithopter is an artificial bird or insect-like aerial vehicle whose flight and lift movements are produced and maintained by flapping wings. The aerodynamical drag forces responsible for the flying movements are generated by the wing attitude and torques applied to its joints. This mechanical system represents a challenging problem because its dynamics consist of MIMO nonlinear equations with couplings in the input variables. For dealing with such a mathematical model, an Active Disturbance Rejection Control-based (ADRC) method is considered. The cited control technique has been studied for almost two decades and its main characteristics are the use of an extended state observer to estimate the nonmeasurable signals of the plant and a state-feedback control law in standard form fed by that observer. However, even today, the application of the basic methodology requires the exact knowledge of the plant’s control gain which is difficult to measure in the case of systems with uncertain parameters. In addition, most of the related works apply the ADRC strategy to Single Input Single Output (SISO) plants. For MIMO systems, the control gain is represented by a square matrix of general entries but most of the reported works consider the simplified case of uncoupled inputs, in which a diagonal matrix is assumed. In this paper, an extension of the ADRC SISO strategy for MIMO systems is proposed. By adopting such a control methodology, the resulting closed-loop scheme exhibits some key advantages: (i) it is robust to parametric uncertainties; (ii) it can compensate for external disturbances and unmodeled dynamics; (iii) even for nonlinear plants, mathematical analysis using Laplace’s approach can be always used; and (iv) it can deal with system’s coupled input variables. A complete mathematical model for the dynamics of the ornithopter wing system is presented. The efficiency of the proposed control is analyzed mathematically, discussed, and illustrated via simulation results of its application in the attitude control of ornithopter wings.

## 1. Introduction

The control of flapping-wing flight vehicles has been receiving increasing attention from the research community over the last few decades [1,2,3,4,5,6,7,8,9]. In this growing field, the literature has reported remarkable progress toward the design, construction, and control of flapping-wing aerial vehicles. Since it is inspired by biological systems such as the flights of birds and insects, it has captured the attention and interest of engineers, roboticists, hobbyists, and even biologists. This interest is also due to the ability of these systems to hover and maneuver quickly [5]. In [10], the authors describe the design and fabrication of a soft robotic dragonfly whose flapping wings are powered by dense dielectric elastomer actuators (DEAs). Some of the advantages raised by the authors are that these aerial robots operated by DEAs demonstrated insect-like resilience and agility. However, a notable drawback is that these flexible actuators require upwards of 500 volts to operate. Notably, this condition presents significant practical challenges for the use of electronic controllers, as the latter require compact and efficient high-voltage power circuits. Nevertheless, many of its attractive motion properties also come at a price. For example, the airflow generated by the wings’ flapping increases the aerodynamics’ complexity around the flier’s control surfaces of the flier [6]. Undoubtedly, these challenges/difficulties need to be taken into account in the design of the controller algorithm.

In [9], the authors were concerned with describing the mathematical model of the ornithopter using the Rayleigh–Ritz method which, according to the reported study, is more robust to wing shape changes and seems to be more suitable for optimization of profile design. Although the authors have not addressed the control problem of the mechanism, the proposed model seems to be computationally efficient and suitable for control purposes, and complex parametric analyses. In [11], the authors present an analysis of the unstable aerodynamics of low-aspect-ratio ellipsoidal wing-flapping ornithopters. In the study reported by these authors, the aerodynamics are analyzed and modeled using three-dimensional simulations by the Computational Fluid Dynamics (CFD) method.

One of the most significant difficulties in the control systems design is obtaining the mathematical description of the system since a modeling error may generate unmodeled dynamics and/or uncertainties in the values of the system’s parameters. The system is then said to be uncertain, as its control is a relevant problem because of its industrial and academic applications. Among the several control strategies for uncertain systems proposed in the literature concerning linear and nonlinear systems, it can be cited the adaptive Backstepping [12], Model Reference Adaptive Control (MRAC) [13], VS-MRAC [14], and others. In addition to the system uncertainties, another fundamental problem in the control system design is the efficient rejection of external disturbance, where some relevant nonlinear control strategies were proposed [15,16,17,18].

In this context, the Active Disturbance Rejection Control (ADRC) strategy emerged [19,20,21,22], which uses an Extended State Observer (ESO) to estimate the unavailable signals of systems, including the nonmodeled dynamics and the external disturbance. Then, these estimates are used in a stabilizing control law, generating a control strategy with simple implementation and robustness properties to external disturbance and nonmodeled dynamics. Due to these advantages, extensions of this control strategy have been developed, allowing its application in a broader class of systems. As an example, in Zheng et al. [23], the ADRC strategy was extended to Multiple Inputs-Multiple Outputs (MIMO) systems being applied in an industrial chemical process. For higher-order systems, in [24], it is proposed the use of a cascaded of the reduced-order estimators, where the estimation and control parameters are maintained for each observer, and the estimates of the parametric values are obtained using a particle swarm optimization procedure according to an established cost function. In Sun et al. [25], an extension of ADRC is proposed for nonminimum phase systems, where a feedforward control assisted by the model is used to assure a null stationary error with a minimum settling time. As can be seen in these and other recent works involving ADRC for SISO plants [21,22,24,26,27,28,29], and also for MIMO plants [30,31], the uncertainties in dynamical parameters are taking into account, despite assuming that the control gains are considered to be known. Therefore, in many practical applications, such as load transportation on mobile robots and/or drones, the values of load mass and the system gravity center vary during the task execution, which causes an uncertainty in the values of the inertia matrix. This matrix strongly influences the control gain [32,33], which also becomes uncertain.

Aiming to solve the problem of an uncertain control gain, an extension of ADRC, namely modified ADRC, was proposed in [34]. The modification consists of inserting a constant gain in series with the system’s output and a filter inserted in parallel with them. Then, aiming to obtain an estimate considering the external disturbance, nonmodeled dynamics, and parametric uncertainty (including the control gain), an ESO is designed, where it is only necessary to know the signal of the control gain.

However, as only the case of SISO (Single Input, Single Output) systems was mathematically analyzed, the control gain was considered a scalar constant. Thus, to MIMO systems, the mathematical analysis of [34] could be applied only if the input was decoupled (or a weak coupling), where the systems could be represented as a set of SISO systems. However, many MIMO systems have a strong coupling between the inputs. In this case, the strategy of considering the MIMO system as a set of SISO systems has not proved to be efficient. ADRC MIMO strategies were also recently proposed in [35,36]. In [35], the authors describe a very interesting application involving trajectory tracking control in a bus-type land vehicle, in an approach called Self-Driving Bus (SDB). In the case of this system, there is a tight coupling between the vehicle lateral error subsystem and the vehicle speed subsystem. The authors then develop ADRC-based decoupling control for a class of general uncertainty MIMO nonlinear systems. In [36], the application of the ADRC method in the control of Continuous Robots (CRs) is discussed. These are a class of robotic systems with a series of connecting units gathered to perform a given task in a continuous motion manner. In this work, the CR of interest is modeled as a Multi-Input Multi-Output (MIMO) system subjected to an external disturbance, and whose internal parameters are considered uncertain. The ADRC MIMO strategy used in the latter work is an extension of the standard ADRC strategy for SISO systems in which the uncertain part of the control matrix gain is included in the generalized disturbance.

This work presents and discusses an extension of the SISO Modified-Plant ADRC (MP-ADRC) to MIMO systems. As part of the analysis, it is shown that all the robustness properties of the standard SISO MP-ADRC are kept unchanged when applied to the MIMO case. Also, an additional advantage is highlighted in what concerns the knowledge of the system’s matrix control gain. Such a priori information is not required since only the signs of its main diagonal entries are needed, which contracts with other ADRC MIMO strategies. In order to illustrate the application of the proposed MIMO MP-ADRC strategy as a case study, the work considers the attitude control problem of an ornithopter wing, which is also addressed by other works in the literature. For example, the Proportional-Integral-Derivative (PID) controller and its variants are the commonly used schemes for flight control in real-world applications [7,8,37,38]. Classical optimal techniques such as Linear Quadratic Regulator (LQR), Linear Quadratic Integrator (LQI), and Model Predictive Control (MPC) are also adopted as control solutions [39,40]. Robust control schemes such as $H_{\infty }$ also appear in literature as a choice. Sliding Mode Control (SMC) is another robust scheme reported in the literature [41]. Active disturbance rejection techniques are also reported in the works [31,42,43,44].

In [3], the authors investigated the hovering performance of a Micromechanical Flying Insect [1] by designing LQR-based feedback control laws. In particular, this work provides a methodology to approximate the time-varying dynamics governed by the aerodynamic forces with a time-invariant mathematical model obtained via averaging theory and a biomimetic parametrization of the wing trajectories. In [45], the proposed controller is based on adaptive backstepping and requires no knowledge of the aerodynamical parameters. The control algorithm can follow a given angular reference trajectory during the flight task only by actuating on the tail deflection. The work reported in [46] discusses the flight stabilization problem of an avian-scale flapping robot with articulated wings. Because of the impact of the fuselage pitch angle influence on the flight altitude, a cascade control scheme is used to control fuselage and tail pitch angles in inner loops and the altitude in the outer one. In both loops, classical PID control laws are used. In the works [7,37], the authors have used feedback linearization plus PD control methods, which are the most common control approaches applied for nonlinear robotics systems.

In [42], authors use two isolated ADRC schemes to stabilize the attitude of the pitch and roll angles. In the proposed control strategy, disturbances and the coupling effects between pitch and roll channels are estimated by an Extended State Observer (ESO) and then compensated in real time. In [44], an interesting control problem is addressed. The work discusses the stabilization problem applied to flapping-wing robots before a take-off phase. The authors propose a control scheme composed of a feedback linearization part and an active Disturbance Rejection Control (ADRC) estimation stage for rejection purposes.

Although the cited ADRC-based schemes have been well documented in the literature, few have explored the control problem from the multivariable systems point of view. In this sense, the present work intends to present a valuable contribution to the state of the art. In summary, this paper intends to present the following contributions:

1. Present a detailed extension of the MP-ADRC method for application in MIMO systems;
2. Discuss and analyze such extension in the attitude control of an ornithopter wing, which corresponds to a relevant and challenging nonlinear control problem;
3. Analyze the results of computational simulation obtained after control implementation, taking into account the detailed dynamic model of the ornithopter wing;
4. Compare the performance of the proposed control scheme with those obtained with the traditional feedforward PD strategy [8,47].

This paper is organized as follows: in Section 2, the problem statement is presented. The computed torque, a traditional control strategy, is presented in Section 3. The standard ADRC and the MP-ADRC for MIMO systems, proposed in this paper, are presented in Section 4 and Section 5, respectively. Section 6 approaches the application of MP-ADRC for MIMO systems in the dynamic model of the ornithopter wing. The simulation results are shown in Section 7. Finally, Section 8 approaches the final conclusions.

## 2. Problem Statement

**Figure 1:**
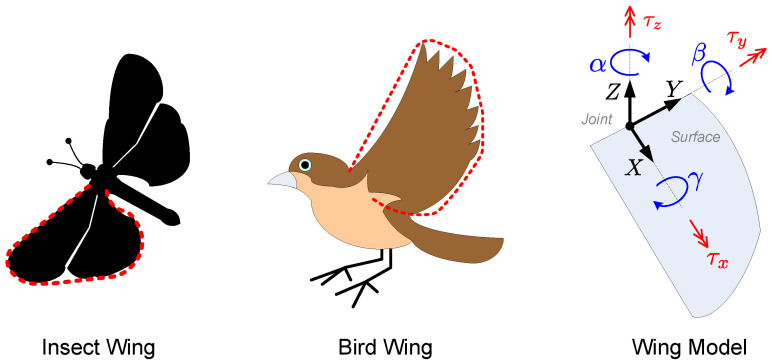
Illustrative images of wing shapes in nature. Ornithopter wing model and its degrees-of-freedom.

In this paper, we consider the attitude control problem of one ornithopter wing, as illustrated in the images of Figure 1. The mechanism of the wing model considered in the current work consists of a three-degrees-of-freedom rotary spherical joint that orientates a flat surface wing according to the joint variables α,β,γ∈IR[rad]. Such rotation angles are defined with respect to their corresponding axis, *Z*, *Y*, and *X*, respectively.

**Figure 2:**
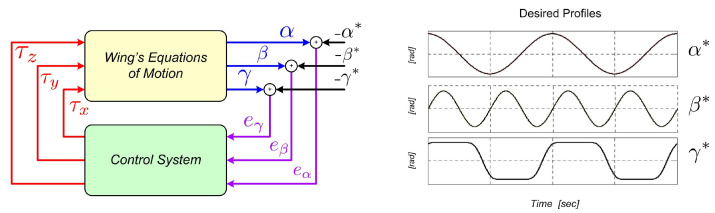
Block diagram of the attitude control system.

The wing motion is performed through the application of the input torques $\tau_{x},\;\tau_{y},\;\tau_{z}\in I\;R\;\left[Nm\right]$ around the $Z_{0}$, $Y_{0}$, and $X_{0}$ axis, respectively. This way, the control objective is to derive convenient mathematical laws for the torque inputs in order to force the joint variables to track some desired angular profiles represented here by $\alpha^{*},\;\beta^{*},\;\gamma^{*}\in I\;R\;\left[rad\right]$. The control system general overview is shown in Figure 2, in which the output error variables $e_{\alpha },\;e_{\beta },\;e_{\gamma }\in I\;R\;\left[rad\right]$ are highlighted together with an illustrative image of the desired angular profiles. Related to the control problem addressed here, the next section presents and discusses the Computed Torque method, which is a traditional control strategy [7,8,37,38].

## 3. Computed Torque Method

As reported in several works in the literature, e.g., [8], the ornithopter wing motion can be represented by the well-known dynamical equation:

(1) $$M\left(q,\dot{q}\right)\ddot{q}+C_{q}\left(q,\dot{q}\right)\dot{q}+N\left(q\right)=\tau +\tau_{v}\;,$$

in which $q={\left[\alpha \;\beta \;\gamma \right]}^{T}$ is the joint vector, $\tau ={\left[\tau_{\alpha }\;\tau_{\beta }\;\tau_{\gamma }\right]}^{T}$ is the torque (input) vector, $\tau_{v}={\left[\tau_{v1}\;\tau_{v2}\;\tau_{v3}\right]}^{T}$ is the vector of aerodynamical forces that act in the wing, $M\left(q,q\right)\in I\;R^{3\times 3}$ is the symmetric positive definite inertia matrix, $C_{q}\left(q,\dot{q}\right)\in I\;R^{3\times 3}$ is the Coriolis matrix, and $N\left(q\right)\in I\;R^{3}$ is the vector of gravity force terms and other generalized forces that act in the wing joints. All these dynamical parameters are detailed in Appendix A. Since $M(q,\dot{q})$ is nonsingular due to its well-known structural property [47], then Equation (1) can be rewritten as

(2) $$\ddot{q}=M^{-1}\left(q,\dot{q}\right)\left[-C_{q}\left(q,\dot{q}\right)\dot{q}-N\left(q\right)+\tau_{v}\right]\;+\;M^{-1}\left(q,\dot{q}\right)\tau \;,$$

or, in the following equivalent compact form

(3) $$\ddot{q}=F\left(q,\dot{q},\tau_{v}\right)+B\left(q,\dot{q}\right)\tau \;.$$

**Figure 3:**
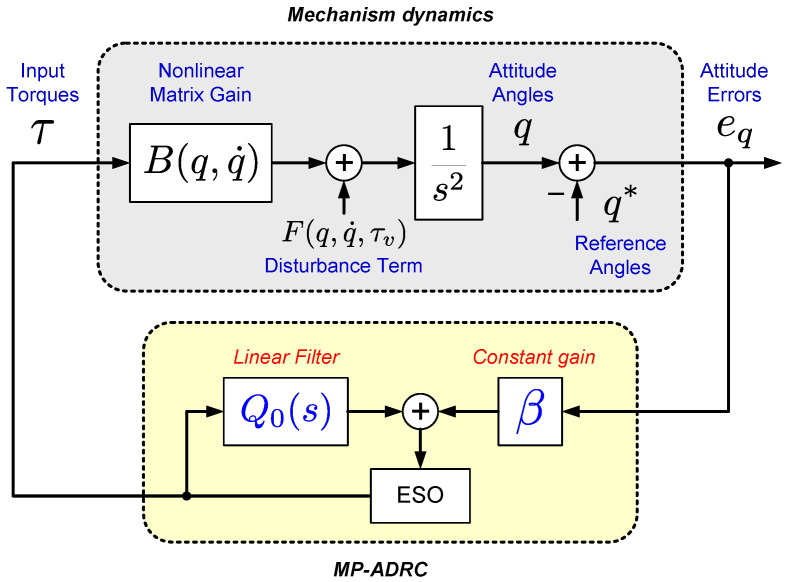
Block diagram of the proposed MIMO MP-ADRC strategy.

From Equation (2) to Equation (3), the description of the dynamics has been conveniently rearranged to represent the set of original equations in an equivalent (still vector) set. This equivalent one now consists of a linear double integrator plant subjected to the vector input terms $F(q,\dot{q},\tau_{v})$ and $B(q,\dot{q})\tau$, as depicted in Figure 3. In the ideal case, considering that all model parameters and variables are known and available, a control law for the torque vector τ could be chosen as follows.

(4) $$\begin{array}{cc} \; & \tau =B{\left(q,\;\dot{q}\right)}^{-1}\left[-K_{1}E-K_{2}\dot{E}-F\left(q,\dot{q},\tau_{v}\right)+{\ddot{q}}^{*}\right]\;, \end{array}$$

(5) $$\begin{array}{cc} \; & E={\left[e_{\alpha }\;e_{\beta }\;e_{\gamma }\right]}^{T}\;,\;e_{\alpha }=\alpha -\alpha^{*}\;,\;e_{\beta }=\beta -\beta^{*}\;,\;e_{\gamma }=\gamma -\gamma^{*}\;, \end{array}$$

(6) $$\begin{array}{cc} \; & q^{*}={\left[\alpha^{*}\;\beta^{*}\;\gamma^{*}\right]}^{T}\;, \end{array}$$

in which $K_{1},\;K_{2}\in I\;R^{3\times 3}$ are diagonal and positive definite matrices with convenient entries. By simply replacing the expression of Equation (4) in Equation (3), one can verify that the closed-loop system assumes the format:

(7) $$\ddot{E}+K_{2}\dot{E}+K_{1}E=0\;,$$

which represents an exponentially stable MIMO error system. However, in many applications, the entries of the vector term $F(q,\dot{q},\tau_{v})$ can involve uncertain parameters, unknown and/or unmodeled dynamics, unmeasurable signals, and external disturbances, which prevent Equation (4) from being implemented directly as it is.

For solving the MIMO control problem concerning ornithopter dynamics, we adopt an extension of the SISO ADRC method proposed in [34]. The main characteristics that make ADRC an attractive control technique for the current challenge are its well-known robustness properties against parametric uncertainties, the unmodeled dynamics of the system’s model, and its ability to reject external disturbances. In the basic SISO ADRC method introduced in [19], and further improved in [21,48,49], the design procedures consider the knowledge of the system’s control coefficient (or simply the control gain) or some nominal value nearby. In [34], such a priori knowledge is avoided, and only the sign of that coefficient is required in the control design. Although the latter method has been proposed for general SISO nonlinear systems, this work exploits an extension for MIMO systems aiming to take advantage of some of its established robustness properties. An illustrative diagram of the proposed MIMO ADRC scheme is also shown in Figure 3.

## 4. The ADRC Method Applied to MIMO Dynamical Systems

Let us consider a class of nonlinear MIMO plants in the following format.

(8) $$\ddot{X}=F\left(X,\dot{X},W\right)+B\tau \;,$$

in which $X={\left[x_{1}\;x_{2}\;x_{3}\right]}^{T}\in I\;R^{3}$ represents the vector of the system’s output variables, $\tau ={\left[\tau_{1}\;\tau_{2}\;\tau_{3}\right]}^{T}\in I\;R^{3}$ represents the vector of the system’s input (control) variables, $F\left(X,\dot{X},W\right)={\left[f_{1}\;f_{2}\;f_{3}\right]}^{T}\in I\;R^{3}$ represents the vector of the system’s nonlinear functions, and $W={\left[w_{1}\;w_{2}\;w_{3}\right]}^{T}\in I\;R^{3}$ is the vector of external disturbances. For illustrative purposes, we can expand Equation (8) to highlight its line expressions:

(9) $$\begin{array}{ccc} {\ddot{x}}_{1} & = & f_{1}\left(X,\dot{X},W\right)+b_{11}\tau_{1}+b_{12}\tau_{2}+b_{13}\tau_{3},\\ {\ddot{x}}_{2} & = & f_{2}\left(X,\dot{X},W\right)+b_{21}\tau_{1}+b_{22}\tau_{2}+b_{23}\tau_{3},\\ {\ddot{x}}_{3} & = & f_{3}\left(X,\dot{X},W\right)+b_{31}\tau_{1}+b_{32}\tau_{2}+b_{33}\tau_{3}, \end{array}$$

in which $b_{ij}\;\left(i,j=1,2,3\right)$ are the constant coefficients for the control variables $\tau_{1},\tau_{2},\tau_{3}\in I\;R$. The control objective is to define convenient laws for $\tau_{1},\tau_{2},\tau_{3}$ that force the system’s output variables $x_{1},x_{2},x_{3}$ to converge asymptotically to the desired bounded reference trajectories $x_{1}^{*},x_{2}^{*},x_{3}^{*}$, respectively. For simplifying notations in Equation (8), the vector $F(X,\dot{X},W)$ will be denoted by F(t) henceforth.

Regarding the general MIMO plant of Equation (8), from Equation (4), it is well-known that for a nonsingular *B* and known F(t), a control law that would achieve the desired output tracking ideally would be given by:

(10) $$\tau =B^{-1}\left[-K_{1}E-K_{2}\dot{E}-F\left(t\right)+{\ddot{X}}^{*}\right]$$

where $E:=X-X^{*}$, $X^{*}={\left[x_{1}^{*}\;x_{2}^{*}\;x_{3}^{*}\right]}^{T}$ is the vector of reference trajectories and $K_{1},K_{2}\in I\;R^{3\times 3}$ are diagonal and positive definite matrix gains. However, in many applications, the generalized disturbance vector F(t) can involve unknown and/or unmodeled dynamics, unmeasurable signals, and external disturbances. In such cases, the control law of Equation (10) can not be directly computed.

### Standard ADRC Design Methodology

In order to overcome the implementation problem of Equation (10), an Extended State Observer (ESO) is designed to estimate F(t) and all the system state variables. The strategy is to define the plant state vector as $\bar{X}$ and insert F(t) as an additional state variable:

(11) $$\bar{X}\left(t\right)=\left[\begin{array}{c} {\bar{X}}_{1}\\ {\bar{X}}_{2}\\ {\bar{X}}_{3} \end{array}\right]=\left[\begin{array}{c} X\\ \dot{X}\\ F(t) \end{array}\right]$$

By assuming that F(t) is a vector of differentiable functions, the system state-space representation assumes the following format:

(12) $$\begin{array}{cc} \; & \dot{\bar{X}}=\underset{A}{\underset{︸}{\left[\begin{array}{ccc} 0 & I & 0\\ 0 & 0 & I\\ 0 & 0 & 0 \end{array}\right]}}\bar{X}+\underset{\bar{B}}{\underset{︸}{\left[\begin{array}{c} 0\\ I\\ 0 \end{array}\right]}}B\tau +\underset{B_{1}}{\underset{︸}{\left[\begin{array}{c} 0\\ 0\\ I \end{array}\right]}}\dot{F}\left(t\right),\\ \; & Y\left(t\right)=\underset{C}{\underset{︸}{\left[\begin{array}{ccc} I & 0 & 0 \end{array}\right]}}\bar{X}, \end{array}$$

in which $0\in I\;R^{3\times 3}$ is the zero matrix and $I\in I\;R^{3\times 3}$ is the identity matrix. Note that in such a configuration, the pair (A,C) is always observable. Thus, the design of an Extended State Observer (ESO), with state $\bar{X}$ is feasible and can be given by:

(13) $$\dot{\hat{\bar{X}}}=\left(A-LC\right)\hat{\bar{X}}+B\tau +LY\left(t\right)$$

where $\hat{\bar{X}}={\left[{\hat{\bar{X}}}_{1}^{T}\;{\hat{\bar{X}}}_{2}^{T}\;{\hat{\bar{X}}}_{3}^{T}\right]}^{T}$ is the estimate of $\bar{X}$ and $L\in I\;R^{9\times 3}$ is the estimator gain matrix. Note, from Equations (12) and (13), that estimation error dynamic $\tilde{\bar{X}}=\bar{X}-\hat{\bar{X}}$ is described by

(14) $$\dot{\tilde{\bar{X}}}=\left(A-LC\right)\tilde{\bar{X}}+B_{1}\dot{F}\left(t\right).$$

Moreover, since Equation (12) is an observable representation, the eigenvalues of A−LC can be placed in predetermined locations on the Left Half-Plane (LHP). In this situation, the system represented by Equation (14) is BIBO, and the estimation error $\tilde{\bar{X}}$ will remain bounded for a bounded $\dot{F}\left(t\right)$, ∀t. Note also that due to the property of pole placement freedom, the error convergence $\tilde{\bar{X}}\to 0$ will be guaranteed in the case $\dot{F}\left(t\right)$ tends to zero. Thus, using the estimated states $\hat{\bar{X}}$ in Equation (10), the following state-feedback control law is proposed:

(15) $$\tau =B^{-1}\left[-K_{1}\left({\hat{\bar{X}}}_{1}-X^{*}\right)-K_{2}\left({\hat{\bar{X}}}_{2}-{\dot{X}}^{*}\right)-{\hat{\bar{X}}}_{3}+{\ddot{X}}^{*}\right].$$

#### Stability Analysis

Let us define the reference trajectory vector as ${\bar{X}}^{*}:={\left[X^{*T},{\dot{X}}^{*T},0\right]}^{T}$ and the tracking error as $\bar{E}:=\bar{X}-{\bar{X}}^{*}$. Then, from Equation (12), the tracking error dynamics can be described by

(16) $$\dot{\bar{E}}=A\bar{E}+\bar{B}B\tau +A{\bar{X}}^{*}+B_{1}\dot{F}-{\dot{\bar{X}}}^{*}\;.$$

Note that the control law τ can be defined like Equation (15), i.e.,

(17) $$\tau =B^{-1}\left(-K\bar{E}+K\tilde{\bar{X}}+{\ddot{X}}^{*}\right)$$

where we define $K:=[K_{1},K_{2},I]$. Then, by replacing Equation (17) into Equation (16), we obtain

(18) $$\dot{\bar{E}}=\left(A-\bar{B}K\right)\bar{E}+\bar{B}K\tilde{\bar{X}}+B_{1}\dot{F}+\bar{B}{\ddot{X}}^{*}+A{\bar{X}}^{*}-{\dot{\bar{X}}}^{*},$$

from which we highlight the following property

(19) $$\bar{B}{\ddot{X}}^{*}+A{\bar{X}}^{*}-{\dot{\bar{X}}}^{*}\;=\;\left[\begin{array}{c} 0\\ I\\ 0 \end{array}\right]{\ddot{X}}^{*}+\left[\begin{array}{ccc} 0 & I & 0\\ 0 & 0 & I\\ 0 & 0 & 0 \end{array}\right]\left[\begin{array}{c} X^{*}\\ {\dot{X}}^{*}\\ 0 \end{array}\right]-\left[\begin{array}{c} {\dot{X}}^{*}\\ {\ddot{X}}^{*}\\ 0 \end{array}\right]\;=\;\left[\begin{array}{c} 0\\ 0\\ 0 \end{array}\right]\;.$$

Thus, by gathering together Equation (14) and the remaining part of Equation (18) as an augmented error vector, then the overall system closed-loop error dynamics results in:

(20) $$\left[\begin{array}{c} \dot{\tilde{\bar{X}}}\\ \dot{\bar{E}} \end{array}\right]=\left[\begin{array}{cc} A-LC & 0\\ \bar{B}K & A-\bar{B}K \end{array}\right]\left[\begin{array}{c} \tilde{\bar{X}}\\ \bar{E} \end{array}\right]+\left[\begin{array}{c} B_{1}\\ B_{1} \end{array}\right]\dot{F}\left(t\right).$$

Since the closed-loop characteristic roots emerge from the eigenvalues of matrices A−LC and $A-\bar{B}K$, these can be placed arbitrarily on the LHP by choosing the entries of both the ESO gain matrix *L* and the controller gain matrix *K* conveniently. Hence, by assuming a bounded $\dot{F}$, which is a reasonable assumption in practice, the following statements hold:

- The resultant closed-loop system is BIBO;
- The tracking error entries of vector $\bar{E}$ are uniformly bounded ∀t;
- In a steady-state regime, $\bar{E}\to 0$ if $\dot{F}\left(t\right)\to 0$.

In the previous approach, note that *B* is required to be a known matrix for implementing the control strategy of Equation (15). If *B* is not known exactly, then it is not possible to implement the ESO of Equation (12) as it is. Thus, the generalized disturbance F(t) estimate can not be guaranteed to succeed, leading to tracking error degradation or instability. The problem concerning the lack of requirement on the knowledge of the *B* matrix is addressed in the next section, in which an extension of the MP-ADRC method is discussed for MIMO systems with uncertain *B* matrix.

## 5. Modified-Plant ADRC (MP-ADRC)

Consider the general class of dynamical systems described by Equation (8). For analysis purposes, let us detach the system linear apart from F(t) so that it can appear explicitly in the plant representation, as follows:

(21) $$\ddot{X}=\underset{F(t)}{\underset{︸}{-A_{1}\dot{X}-A_{0}X+G\left(t\right)}}+B\tau ,$$

where $A_{1},\;A_{0}\in I\;R^{3\times 3}$ are constant matrices and $G\left(t\right)\in I\;R^{3}$ is the generalized disturbance vector that includes the nonlinear dynamics and the external disturbances. Before continuing to develop the MP-ADRC, the following assumptions are considered:

**Assumption** **1.** *The entries $g_{i}\left(t\right),\;\left(i=1,2,3\right)$ of the generalized disturbance vector G(t) and their first-order derivatives ${\dot{g}}_{i}\left(t\right)$ are bounded by known constants $g_{p},g_{d}>0\in I\;R$, that is,*

(22) $$\begin{array}{cc} & g_{p}\;>\;{∥G\left(t\right)∥}_{\infty }\;, \end{array}$$

(23) $$\begin{array}{cc} & g_{d}\;>\;{∥\dot{G}\left(t\right)∥}_{\infty }\;. \end{array}$$

**Assumption** **2.** *The constant entries of $A_{1}$, $A_{0}$, and B are uncertain, but with known constant upper and lower bounds $M_{0}>0\in I\;R$ and $M_{b}>0\in I\;R$, respectively, given by:*

(24) $$M_{0}\;>\;\sigma_{M}\left(A_{0}\right)\;,\;M_{b}\;<\;\sigma_{m}\left(B\right)\;,$$

*where $\sigma_{m}\left(.\right)$, and $\sigma_{M}\left(.\right)$ stand for the lowest and the largest matrix singular value, respectively.*

**Assumption** **3.** *The matrix B is nonsingular, dominant diagonal and the signs of its main diagonal entries are known.*

As will be shown in the next section, the Assumption 1 is critical for choosing the bandwidth of the state estimator, which should be greater than the bandwidth of G(t). Assumption 2 allows us to consider a more general class of plants with a complete set of uncertain parameters, including the input coefficient (also known as control gain). Assumption 3 is feasible since several mechanical systems have at least a main actuation on each degree of freedom.

### 5.1. Proposed Methodology

The idea of the modified ADRC scheme is to perform a structural change in the plant of Equation (8) input/output description in order to obtain a new dynamical system with some desired characteristics. As will be shown, by adopting such a modification, both controller and ESO designs are simplified in terms of fewer requirements on the knowledge of plant parameters. The original system tracking objective is kept unchanged together with the well-known stability and convergence properties.

**Figure 4:**
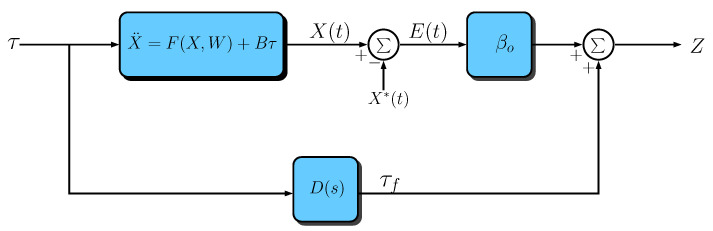
Block diagram of the modified approach.

As can be seen in the diagram of Figure 4, the plant modification is performed by a constant gain matrix $\beta_{o}\in I\;R^{3\times 3}$ that is introduced in series with the plant output error and a diagonal matrix of second-order filters D(s) in parallel with them. These filters are given by:

(25) $$D\left(s\right)=\left[\begin{array}{ccc} d(s) & 0 & 0\\ 0 & d(s) & 0\\ 0 & 0 & d(s) \end{array}\right]\;,$$

in which,

(26) $$d\left(s\right)=\frac{s}{{\left(s+p\right)}^{2}}\;=\;\frac{s}{s^{2}+\alpha_{1}s+\alpha_{0}}\;,\;p>0\in I\;R\;,$$

is a stable filter (poles with negative real parts).

Based on the diagram of Figure 4, it can be concluded that:

(27) $$\begin{array}{ccc} Z(t) & = & \beta_{o}E\left(t\right)+\tau_{f}\left(t\right)\;, \end{array}$$

(28) $$\begin{array}{ccc} E(t) & = & X\left(t\right)-X^{*}\left(t\right)\;, \end{array}$$

(29) $$\begin{array}{ccc} {\ddot{\tau }}_{f} & = & -\Lambda_{1}{\dot{\tau }}_{f}-\Lambda_{0}\tau_{f}+\dot{\tau }\;, \end{array}$$

where $\Lambda_{1}=\alpha_{1}I$ and $\Lambda_{0}=\alpha_{0}I$. Note from Equation (26) that $\alpha_{1}=2p$ and $\alpha_{0}=p^{2}$. By differentiating Equation (27) twice with respect to time and using the expressions in Equations (21) and (28), one can conclude that

(30) $$\ddot{Z}=\beta_{o}\left(-A_{1}\dot{X}-A_{0}X+G\left(t\right)+B\tau -{\ddot{X}}^{*}\right)+{\ddot{\tau }}_{f}.$$

Since $\tau_{f}=Z-\beta_{o}E$, then, from Equation (29), we have that

(31) $${\ddot{\tau }}_{f}=-\Lambda_{1}\left(\dot{Z}-\beta_{o}\dot{E}\right)-\Lambda_{0}\left(Z-\beta_{o}E\right)+\dot{\tau }.$$

Thus, by replacing the expression of Equation (31) in Equation (30), we obtain the modified plant equation, now written in terms of the new output error vector *Z*, the new generalized disturbance vector Ω(t) and the new control vector $\dot{\tau }$:

(32) $$\begin{array}{cc} \; & \ddot{Z}+\Lambda_{1}\dot{Z}+\Lambda_{0}Z=\Omega \left(t\right)+\dot{\tau }\;, \end{array}$$

(33) $$\begin{array}{cc} \; & \Omega \left(t\right)=\beta_{o}\left(-A_{1}\dot{X}-A_{0}X+G\left(t\right)+B\tau -{\ddot{X}}^{*}+\Lambda_{1}\dot{E}+\Lambda_{0}E\right)\;. \end{array}$$

Note, from Equations (32) and (33), that the input matrix *B* is inserted into the new generalized disturbance Ω(t) and the new input signal is $\dot{\tau }$, which has the identity matrix *I* as its coefficient. Therefore, if vector Ω(t) was known, then it would be enough to choose the trajectory tracking control law as $\dot{\tau }=-\Omega \left(t\right)$. This particular choice could make the closed-loop system of Equation (32) become exponentially stable. However, as Ω(t) is an uncertain vector, then an ESO is suggested for estimating it. Thus, defining ${\bar{Z}}_{1}=Z$, ${\bar{Z}}_{2}=\dot{Z}$ and ${\bar{Z}}_{3}=\Omega \left(t\right)$ as state variables, the Equation (32) can be describe by

(34) $$\begin{array}{cc} \; & \left[\begin{array}{c} {\dot{\bar{Z}}}_{1}\\ {\dot{\bar{Z}}}_{2}\\ {\dot{\bar{Z}}}_{3} \end{array}\right]=\underset{\bar{A}}{\underset{︸}{\left[\begin{array}{ccc} 0 & I & 0\\ -\Lambda_{0} & -\Lambda_{1} & I\\ 0 & 0 & 0 \end{array}\right]}}\underset{\bar{Z}}{\underset{︸}{\left[\begin{array}{c} {\bar{Z}}_{1}\\ {\bar{Z}}_{2}\\ {\bar{Z}}_{3} \end{array}\right]}}+\underset{\bar{B}}{\underset{︸}{\left[\begin{array}{c} 0\\ I\\ 0 \end{array}\right]}}\dot{\tau }+\underset{\Gamma }{\underset{︸}{\left[\begin{array}{c} 0\\ 0\\ I \end{array}\right]}}\dot{\Omega }\left(t\right)\\ \; & Z=\underset{\bar{C}}{\underset{︸}{\left[\begin{array}{ccc} I & 0 & 0 \end{array}\right]}}\bar{Z}. \end{array}$$

**Remark** **1.** *Note that ESO’s implementation of Equation (34) does not require the value of the input gain matrix B of the plant, which contrasts with other ADRC methodologies in the literature. This is due to the fact that now B is inserted within the disturbance* Ω*, as described by Equation (33). In this approach, B will be estimated together with* Ω*. This is the main feature that gives the MP-ADRC method a higher level of robustness to uncertainties in B.*

In order to facilitate the estimator design, a state transformation matrix *T* is used:

(35) $$\bar{Z}\;=\;T{\bar{Z}}_{o}\;,\;T\;=\;\left[\begin{array}{ccc} I & 0 & 0\\ -\Lambda_{1} & I & 0\\ 0 & 0 & I \end{array}\right]\;,$$

where ${\bar{Z}}_{o}^{T}=\left[\begin{array}{ccc} {\bar{Z}}_{o1}^{T} & {\bar{Z}}_{o2}^{T} & {\bar{Z}}_{o3}^{T} \end{array}\right]$ is the state vector in the canonical observable form. Note that $Z={\bar{Z}}_{1}={\bar{Z}}_{o1}$ and $\Omega \left(t\right)={\bar{Z}}_{3}={\bar{Z}}_{o3}$. Then, it is known that

(36) $$\begin{array}{cc} \; & {\dot{\bar{Z}}}_{o}=\underset{{\bar{A}}_{o}}{\underset{︸}{\left[\begin{array}{ccc} -\Lambda_{1} & I & 0\\ -\Lambda_{0} & 0 & I\\ 0 & 0 & 0 \end{array}\right]}}{\bar{Z}}_{o}+\underset{{\bar{B}}_{o}}{\underset{︸}{\left[\begin{array}{c} 0\\ I\\ 0 \end{array}\right]}}\dot{\tau }+\underset{\Gamma_{o}}{\underset{︸}{\left[\begin{array}{c} 0\\ 0\\ I \end{array}\right]}}\dot{\Omega }\left(t\right)\;,\\ \; & Z=\underset{{\bar{C}}_{o}}{\underset{︸}{\left[\begin{array}{ccc} I & 0 & 0 \end{array}\right]}}{\bar{Z}}_{o}\;, \end{array}$$

in which ${\bar{A}}_{o}=T^{-1}\bar{A}T$, ${\bar{B}}_{o}=T^{-1}\bar{B}$, $\Gamma_{o}=T^{-1}\Gamma$ and ${\bar{C}}_{o}=\bar{C}T$. Thus, the ESO equation in canonical observable form Equation (36) is given by

(37) $${\dot{\hat{\bar{Z}}}}_{o}=\left({\bar{A}}_{o}-\bar{L}{\bar{C}}_{o}\right){\hat{\bar{Z}}}_{o}+{\bar{B}}_{o}\dot{\tau }+\bar{L}Z\;,$$

where

(38) $${\bar{A}}_{o}-\bar{L}{\bar{C}}_{o}=\left[\begin{array}{ccc} -(\Lambda_{1}+{\bar{L}}_{1}) & I & 0\\ -(\Lambda_{0}+{\bar{L}}_{2}) & 0 & I\\ -{\bar{L}}_{3} & 0 & 0 \end{array}\right]\;,$$

${\hat{\bar{Z}}}_{o}$ is the estimate of ${\bar{Z}}_{o}$, and ${\bar{L}}^{T}=\left[\begin{array}{ccc} {\bar{L}}_{1}^{T} & {\bar{L}}_{2}^{T} & {\bar{L}}_{3}^{T} \end{array}\right]$ is the ESO gain matrix that is responsible for allocating the closed-loop characteristics roots (or poles) of the estimator. The entries of $\bar{L}$ can be computed by solving the matrix polynomial equation

(39) $$s^{3}I+\left(\Lambda_{1}+{\bar{L}}_{1}\right)s^{2}+\left(\Lambda_{0}+{\bar{L}}_{2}\right)s+{\bar{L}}_{3}={\left(s+\omega_{0}\right)}^{3}I\;,$$

being $\omega_{0}\in I\;R$ a positive design constant that defines the ESO characteristics roots and also defines the size of its bandwidth. Then, one can conclude that

(40) $$\begin{array}{ccc} {\bar{L}}_{1} & = & 3\omega_{0}I-\Lambda_{1}\;, \end{array}$$

(41) $$\begin{array}{ccc} {\bar{L}}_{2} & = & 3\omega_{0}^{2}I-\Lambda_{0}\;, \end{array}$$

(42) $$\begin{array}{ccc} {\bar{L}}_{3} & = & \omega_{0}^{3}I\;. \end{array}$$

Hence, the control law to the modified plant of Equation (32) can be described now as a function of ${\hat{\bar{Z}}}_{o3}$, that is the estimate of Ω(t):

(43) $$\dot{\tau }=-{\hat{\bar{Z}}}_{o3}.$$

Note that the convergence analysis of ${\hat{\bar{Z}}}_{o3}$ to generalized disturbance Ω(t) is essential to verify the stability of the closed loop. This analysis is performed in the next section.

### 5.2. ESO Convergence Analysis

From Equations (36) and (37), it can be concluded that the estimation error equation is described by

(44) $$\begin{array}{cc} \; & \dot{\tilde{\bar{Z}}}=\left({\bar{A}}_{o}-\bar{L}{\bar{C}}_{o}\right)\tilde{\bar{Z}}+\Gamma_{0}\dot{\Omega }\\ \; & E_{\Omega }=\Omega \left(t\right)-{\hat{\bar{Z}}}_{o3}=\left[\begin{array}{ccc} 0 & 0 & I \end{array}\right]\tilde{\bar{Z}} \end{array}$$

where $\tilde{\bar{Z}}={\bar{Z}}_{o}-{\hat{\bar{Z}}}_{o}$. In order to write the estimation error equation in the frequency domain, it is convenient to express Equation (44) in controllable form. Then, considering the transformation

(45) $$\tilde{\bar{Z}}=\underset{T_{c}}{\underset{︸}{\left[\begin{array}{ccc} 0 & 0 & I\\ 0 & I & \Lambda_{1}+{\bar{L}}_{1}\\ I & \Lambda_{1}+{\bar{L}}_{1} & \Lambda_{0}+{\bar{L}}_{2} \end{array}\right]}}{\tilde{\bar{Z}}}_{c}$$

in which ${\tilde{\bar{Z}}}_{c}^{T}=\left[\begin{array}{ccc} {\tilde{\bar{Z}}}_{c_{1}}^{T} & {\tilde{\bar{Z}}}_{c_{2}}^{T} & {\tilde{\bar{Z}}}_{c_{3}}^{T} \end{array}\right]$, the representation of Equation (44) in controllable form is

(46) $$\begin{array}{cc} \; & {\dot{\tilde{\bar{Z}}}}_{c}=\left[\begin{array}{ccc} -(\Lambda_{1}+{\bar{L}}_{1}) & -(\Lambda_{0}+{\bar{L}}_{2}) & -L_{3}\\ I & 0 & 0\\ 0 & I & 0 \end{array}\right]{\tilde{\bar{Z}}}_{c}+\left[\begin{array}{c} I\\ 0\\ 0 \end{array}\right]\dot{\Omega }\\ \; & E_{\Omega }=\left[\begin{array}{ccc} I & {\bar{L}}_{1}+\Lambda_{1} & {\bar{L}}_{2}+\Lambda_{0} \end{array}\right]{\tilde{\bar{Z}}}_{c}. \end{array}$$

From Equation (46), one can conclude that

$${\dddot{\tilde{\bar{Z}}}}_{c_{3}}=-\left(\Lambda_{1}+{\bar{L}}_{1}\right){\ddot{\tilde{\bar{Z}}}}_{c_{3}}-\left(\Lambda_{0}+{\bar{L}}_{2}\right){\dot{\tilde{\bar{Z}}}}_{c_{3}}-{\bar{L}}_{3}{\tilde{\bar{Z}}}_{c_{3}}+\dot{\Omega },$$

$$E_{\Omega }={\ddot{\tilde{\bar{Z}}}}_{c_{3}}+\left(\Lambda_{1}+{\bar{L}}_{1}\right){\dot{\tilde{\bar{Z}}}}_{c_{3}}+\left(\Lambda_{0}+{\bar{L}}_{2}\right){\tilde{\bar{Z}}}_{c_{3}}.$$

Therefore, in the frequency domain, from Equations (40)–(42), we have that

(47) $$\begin{array}{cc} \; & E_{\Omega }=\left[\frac{s^{3}+3\omega_{0}s^{2}+3\omega_{0}^{2}s}{s^{3}+3\omega_{0}s^{2}+3\omega_{0}^{2}s+\omega_{0}^{3}}\right]\Omega \;. \end{array}$$

As can be seen from Equation (44), $E_{\Omega }=\Omega -{\hat{\bar{Z}}}_{o3}$ and then, after a few algebraic manipulations, the expression in Equation (47) simplifies to

(48) $${\hat{\bar{Z}}}_{o3}\left(t\right)=\underset{H(s)}{\underset{︸}{\left[\frac{\omega_{0}^{3}}{{\left(s+\omega_{0}\right)}^{3}}\right]}}\Omega \left(t\right),$$

in which emerges the scalar transfer function H(s) that represents the dynamical relation between the entries of the generalized disturbance Ω(t) and their estimates within ${\hat{\bar{Z}}}_{o3}$. In the frequency domain, due to the low-pass behavior of H(s) in Equation (48), its modulus always lies on the interval (0;1]. This means that if $\omega_{0}$ is chosen with a sufficiently large value greater than the highest frequency of Ω(t), then Equation (48) can be approximated by ${\hat{\bar{Z}}}_{o3}\left(t\right)\approx \Omega \left(t\right)$.

**Remark** **2.** *It is important to mention that the adoption of both time and frequency domain representations in Equations (47) and (48) is only for analysis purposes. Moreover, in cases where Ω(t) involves nonlinear functions of the plant state variables, the definition of an Ω(s) in the frequency domain may not be consistent. Thus, as we want to analyze the amplitude of the estimation error signal in Equation (47) without losing generality, we believe that, in this work, this is a more appropriate mathematical formalism to represent the input/output relationships.*

### 5.3. Closed-Loop Stability and Tracking Analysis

By assuming that $\omega_{0}$ in Equation (48) is chosen conveniently, let us define the variable $c_{0}\in \left(0;1\right]$ for representing the value of the equivalent gain of |H(jω)| computed in some frequency ω, [rad/s] within the range $\left[0;\omega_{0}\right)$. Such a definition is introduced here only for analysis since we aim to focus on the magnitude behavior of the error system in a steady-state regime. For this purpose, we have that

(49) $$c_{0}\;=\;\left|H\left(j\omega \right)\right|.$$

Thus, based on Equation (49), we can rewrite the control law in Equation (43) as

(50) $$\dot{\tau }=-c_{0}\Omega \left(t\right).$$

By replacing Equation (33) in Equation (50), we have that

(51) $$\dot{\tau }=-c_{0}\beta_{o}\left(-A_{1}\dot{X}-A_{0}X+G\left(t\right)+B\tau -{\ddot{X}}^{*}+\Lambda_{1}\dot{E}+\Lambda_{0}E\right)$$

Therefore, using Equations (21) and (29) it can be concluded that

(52) $$\dot{\tau }=-c_{0}\beta_{o}\left(\ddot{E}+\Lambda_{1}\dot{E}+\Lambda_{0}E\right)\;=\;-c_{0}\beta_{o}\left(\ddot{E}+\alpha_{1}\dot{E}+\alpha_{0}E\right)\;.$$

From Equation (26), the control law (52) can be described, in frequency domain, by

(53) $$\tau =-\left[\frac{c_{0}\beta_{o}{\left(s+p\right)}^{2}}{s}\right]E.$$

**Figure 5:**
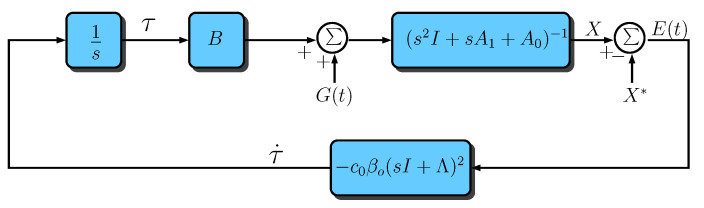
Block diagram of the closed-loop system.

Note, from Equations (53) and (21), that the closed-loop system can be represented by Figure 5, where Λ=pI.

**Figure 6:**
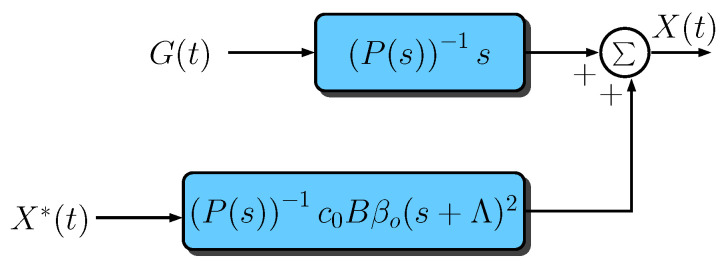
The simplified block diagram of the closed-loop system.

By rearranging the blocks in Figure 5 to place the signal X(t) as output and the signals G(t) and $X^{*}\left(t\right)$ as inputs, the resulting block diagram assumes the format of that one depicted in Figure 6, where

(54) $$P\left(s\right)=s^{3}I+\left(A_{1}+c_{0}B\beta_{o}\right)s^{2}+\left(A_{0}+c_{0}B\beta_{o}\Lambda_{1}\right)s+c_{0}B\beta_{o}\Lambda_{0}\;.$$

By considering the Assumption 2, then $c_{0}$, $\beta_{o}$, $\Lambda_{1}$, and $\Lambda_{2}$ can be chosen such that $\sigma_{m}\left(c_{0}B\beta_{o}\right)\gg M_{1}$ and $\sigma_{m}\left(\Lambda_{1}c_{0}B\beta_{o}\right)\gg M_{0}$. In this case,

(55) $$P\left(s\right)\approx s^{3}I+c_{0}B\beta_{o}s^{2}+c_{0}B\beta_{o}\Lambda_{1}s+c_{0}B\beta_{o}\Lambda_{0}.$$

By recalling that $\Lambda_{1}=\alpha_{1}I=2pI$ and $\Lambda_{0}=\alpha_{0}I=p^{2}I$, it follows that

(56) $$P\left(s\right)\approx s^{3}I+c_{0}B\beta_{o}{\left(sI+pI\right)}^{2}.$$

Moreover, if $\sigma_{m}\left(c_{0}B\beta_{o}\right)\gg \sigma_{M}\left(\Lambda \right)$, then

(57) $$P\left(s\right)\approx \left(sI+c_{0}B\beta_{o}\right){\left(sI+pI\right)}^{2}.$$

Note that by considering the Assumption 3 and the Gershgorin Circle Theorem [50], $\beta_{o}$ always can be chosen to place the eigenvalues of $-c_{0}B\beta_{o}$ on the Left Half-Plane (LHP). In this case, the closed-loop system will be asymptotically stable. Moreover, it can be concluded, from Equation (57) and Figure 3, that

(58) $$X\left(t\right)=\left[{\left(P(s)\right)}^{-1}s\right]G\left(t\right)+\left[{\left(P(s)\right)}^{-1}c_{0}B\beta_{o}{\left(sI+pI\right)}^{2}\right]X^{*}\left(t\right).$$

Then, by replacing Equation (57) in Equation (58), we have that

(59) $$X\left(t\right)=\left[{\left(P(s)\right)}^{-1}s\right]G\left(t\right)+\left[{\left(sI+c_{0}B\beta_{o}\right)}^{-1}c_{0}B\beta_{o}\right]X^{*}\left(t\right).$$

Therefore,

(60) $$E\left(t\right)=\left[{\left(P(s)\right)}^{-1}s\right]G\left(t\right)+\left[{\left(sI+c_{0}B\beta_{o}\right)}^{-1}c_{0}B\beta_{o}-I\right]X^{*}\left(t\right).$$

Thus, one can conclude, considering the Assumption 1, that ||E(t)|| tends to a residual set which can be reduced with the increase of $\left|\right|c_{0}B\beta_{o}{\left|\right|}_{\infty }$. In addition, E(t)→0 if $G\left(t\right),X^{*}\left(t\right)$ tend to be constant values. The following theorem summarizes the obtained result.

**Theorem** **1.** Consider the trajectory tracking control problem of the system class described by Equation (8). Defining $X^{*}$ as the desired reference trajectory and assuming that the Assumptions 1 and 2 hold, then if the filter parameter p and the matrix $\beta_{o}$ are chosen such that

- *all eigenvalues of −Λ and $-c_{0}B\beta_{o}$ belong to the LHP;*
- *$\sigma_{m}\left(c_{0}B\beta_{o}\right)\gg M_{1}$, $\sigma_{m}\left(\Lambda_{1}c_{0}B\beta_{o}\right)\gg M_{0}$, $\sigma_{m}\left(c_{0}B\beta_{o}\right)\gg \sigma_{M}\left(\Lambda \right)$,*

*then the control law of Equation (43) and Extended State Observer (ESO) with gains described in Equation (40), Equations (41) and (42) assure that*

- *E(t) tends to a residual set that can be reduced by increasing $\left|\right|c_{0}B\beta_{o}\left|\right|$;*
- E(t)→0 if $G\left(t\right),X^{*}\left(t\right)$ tend to a constant value.

**Remark** **3.** *The mathematical developments that result in Theorem 1 were applied in a MIMO system composed of three second-order equations. This system was chosen to facilitate the analysis understanding. However, all the steps can be easily extended to systems with a higher number of equations. It is well known that many physical MIMO systems can be mathematically described by n second-order equations, which shows the generality of the results discussed in the present work. For MIMO systems of general order, the design procedures of the modified ADRC technique can be suitably applied; however, they will require some adjustments in the stability-proof mechanism. This will be left as a proposal for future work.*

**Remark** **4.** *The occurrence of delays in the closed-loop system is a practical reality in control systems. As a general consensus in the literature, delay is considered an unmodeled dynamic. Although we have not emphasized this in the current analysis, from a theoretical point of view, one of the characteristics of the ADRC method is the ability to deal with this type of dynamical characteristic. We believe, to the best of our knowledge, that the developments around this analysis can be driven by using the theory presented here in this work together with the Padé approximation Function Theory. In fact, this issue needs to be investigated more closely, which could lead to extensive mathematical developments, additional simulations, and experiments. This topic is not addressed in the current work, by believing that the presentation of such an analysis is not trivial. In this way, we judge it will be more convenient to deal with such a rigorous and complete analysis in future work.*

## 6. Application—Attitude Control of an Ornithopter Wing

In this section, the modified ADRC technique, discussed in the previous sections, is applied to the attitude control problem of an ornithopter wing with a dynamical model described by Equation (1).

### 6.1. System Model

**Figure 7:**
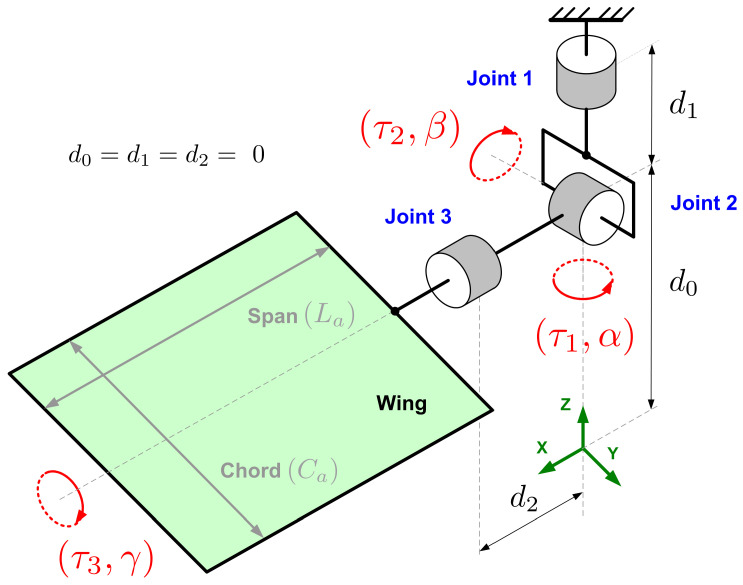
Schematic of an ornithopter wing and its degrees of freedom.

Since the mechanism’s motions behave like a spherical joint, its attitude control can be defined as a tracking problem for the output angles α,β,γ. For the wing motion description, three parameters are introduced: the mass *m*, the chord $C_{a}$, and the span $L_{a}$, as depicted in Figure 7. The dynamical model for the wing motion is described by (1) (see Appendix A for more details).

### 6.2. Control Definition

Since $M(q,\dot{q})$ is a nonsingular matrix, then Equation (1) can be rewritten as

(61) $$\ddot{q}=M^{-1}\left(-C_{q}\left(q,\dot{q}\right)\dot{q}-N\left(q\right)+\tau +\tau_{v}\right)\;=\;\bar{F}\left(q,\dot{q}\right)+M^{-1}\left(\tau +\tau_{v}\right)\;,$$

in which we define

(62) $$\bar{F}{\left(q,\dot{q}\right)}^{T}=\left[{\bar{f}}_{1}\left(q,\dot{q}\right)\;,{\bar{f}}_{2}\left(q,\dot{q}\right)\;,{\bar{f}}_{3}\left(q,\dot{q}\right)\right]=M^{-1}\left(-C_{q}\left(q,\dot{q}\right)\dot{q}-N\left(q\right)\right)\;,$$

and then,

(63) $$\left[\begin{array}{c} {\ddot{q}}_{1}\\ {\ddot{q}}_{2}\\ {\ddot{q}}_{3} \end{array}\right]=\left[\begin{array}{c} {\bar{f}}_{1}\left(q,\dot{q}\right)\\ {\bar{f}}_{2}\left(q,\dot{q}\right)\\ {\bar{f}}_{3}\left(q,\dot{q}\right) \end{array}\right]+M^{-1}\left(\tau +\tau_{v}\right)\;.$$

By noticing that the ornithopter wing dynamics of Equation (63) is similar to Equation (9), with $B=M^{-1}$, then the proposed MP-ADRC scheme of the previous sections can be applied here directly. In fact, after choosing the design parameters *p* of Equation (26), $\beta_{o}$ (28), $\bar{L}$ (40)–(42) properly, one can directly implement Equation (29) for filters, (37) for the ESO and (43) for the control law τ. In this case, the tracking error is defined as

(64) $$e=q-q_{d}\;,$$

in which the reference trajectory is now given in terms of the desired angle vector $q_{d}$, namely

(65) $$q_{d}={\left[\alpha_{r},\;\beta_{r},\;\gamma_{r}\right]}^{T}\;.$$

Thus, based on the developments of the previous sections and on the similarity of systems in Equations (63) and (9), we highlight in the following the final expressions, in expanded form, for summarizing the proposed control system.

For the filter:
  (66) $$\tau_{f}=\left(\frac{s}{s^{2}+\alpha_{1}s+\alpha_{0}}\right)\tau \;,$$
For the auxiliary error:
  (67) $$Z={\left[\begin{array}{ccc} Z_{1} & Z_{2} & Z_{3} \end{array}\right]}^{T}=\beta_{o}\;e+\tau_{f}\;.$$
For the ESOs:
  (a) Subsystem α:
    (68) $$\begin{array}{ccc} {\dot{\hat{Z}}}_{\alpha 1} & = & {\hat{Z}}_{\alpha 2}+L_{1}^{*}\;e_{y\alpha }\;,\\ {\dot{\hat{Z}}}_{\alpha 2} & = & -\alpha_{0}{\hat{Z}}_{\alpha 1}-\alpha_{1}{\hat{Z}}_{\alpha 2}+{\hat{Z}}_{\alpha 3}+{\dot{\tau }}_{\alpha }+L_{2}^{*}\;e_{y\alpha }\;,\\ {\dot{\hat{Z}}}_{\alpha 3} & = & L_{3}^{*}\;e_{y\alpha }\;, \end{array}$$
    (69) $$\begin{array}{ccc} e_{y\alpha } & = & Z_{1}-{\hat{Z}}_{\alpha 1}\;. \end{array}$$
  (b) Subsystem β:
    (70) $$\begin{array}{ccc} {\dot{\hat{Z}}}_{\beta 1} & = & {\hat{Z}}_{\beta 2}+L_{1}^{*}\;e_{y\beta }\;,\\ {\dot{\hat{Z}}}_{\beta 2} & = & -\alpha_{0}{\hat{Z}}_{\beta 1}-\alpha_{1}{\hat{Z}}_{\beta 2}+{\hat{Z}}_{\beta 3}+{\dot{\tau }}_{\beta }+L_{2}^{*}\;e_{y\beta }\;,\\ {\dot{\hat{Z}}}_{\beta 3} & = & L_{3}^{*}\;e_{y\beta }\;, \end{array}$$
    (71) $$\begin{array}{ccc} e_{y\beta } & = & Z_{2}-{\hat{Z}}_{\beta 1}\;. \end{array}$$
  (c) Subsystem γ:
    (72) $$\begin{array}{ccc} {\dot{\hat{Z}}}_{\gamma 1} & = & {\hat{Z}}_{\gamma 2}+L_{1}^{*}\;e_{y\gamma }\;,\\ {\dot{\hat{Z}}}_{\gamma 2} & = & -\alpha_{0}{\hat{Z}}_{\gamma 1}-\alpha_{1}{\hat{Z}}_{\gamma 2}+{\hat{Z}}_{\gamma 3}+{\dot{\tau }}_{\gamma }+L_{2}^{*}\;e_{y\gamma }\;,\\ {\dot{\hat{Z}}}_{\gamma 3} & = & L_{3}^{*}\;e_{y\gamma }\;, \end{array}$$
    (73) $$\begin{array}{ccc} e_{y\gamma } & = & Z_{3}-{\hat{Z}}_{\gamma 1}\;. \end{array}$$
    (74) $$L_{1}^{*}=3\omega_{o}-\alpha_{1}\;,\;L_{2}^{*}=3\omega_{o}^{2}-\alpha_{0}\;,\;L_{3}^{*}=\omega_{o}^{3}\;.$$
For the control laws:
  (75) $$\tau_{\alpha }=-\;\int {\hat{Z}}_{\alpha 3}\;dt\;,\;\tau_{\beta }=-\;\int {\hat{Z}}_{\beta 3}\;dt\;,\;\tau_{\gamma }=-\;\int {\hat{Z}}_{\gamma 3}\;dt\;.$$

In the next section, numerical simulations are performed to illustrate the application of the proposed strategy. The obtained results are presented and discussed after comparisons with other classical control methods.

## 7. Simulation Results

In this section, the performance of the modified ADRC of Equations (64)–(75) is compared with a nonlinear version of the augmented PD control strategy [47] described by

(76) $$\tau =M\left(q_{d},{\dot{q}}_{d}\right){\ddot{q}}_{d}+C_{q}\left(q_{d},{\dot{q}}_{d}\right){\dot{q}}_{d}+N\left(q_{d}\right)-K_{v}\;\left(\dot{q}-{\dot{q}}_{d}\right)-K_{p}\;\left(q-q_{d}\right)\;.$$

**Figure 8:**
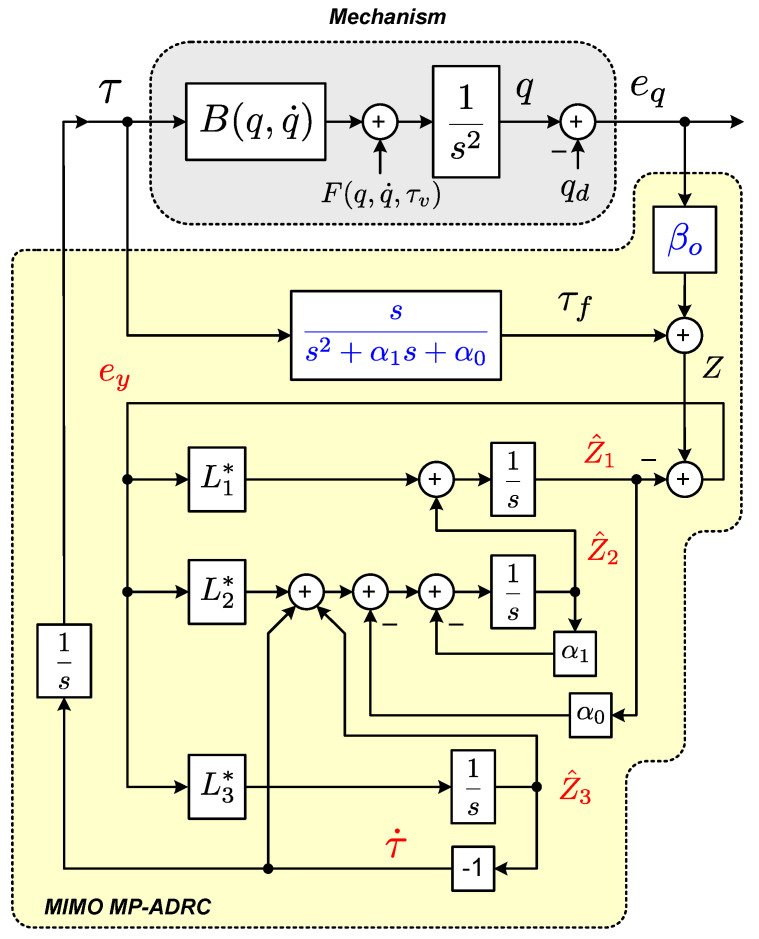
Block diagram of the proposed MIMO MP-ADRC strategy and its implementation.

In addition to the equations explained in the previous section, Figure 8 shows a compact-form block diagram that illustrates the implementation of the proposed method.

The comparison is performed by changing some dynamical parameter values of the ornithopter wing model to mimic some sorts of parametric uncertainties. This is performed in two different simulation trials, as shown in Table 1. The complete set of model parameters and matrices can be found in Appendix A.

**Figure 9:**
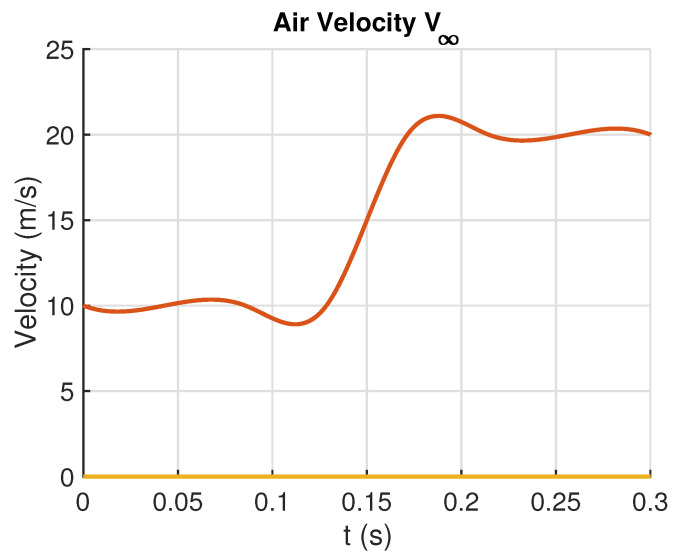
Wind velocity profile used in simulations.

**Figure 10:**
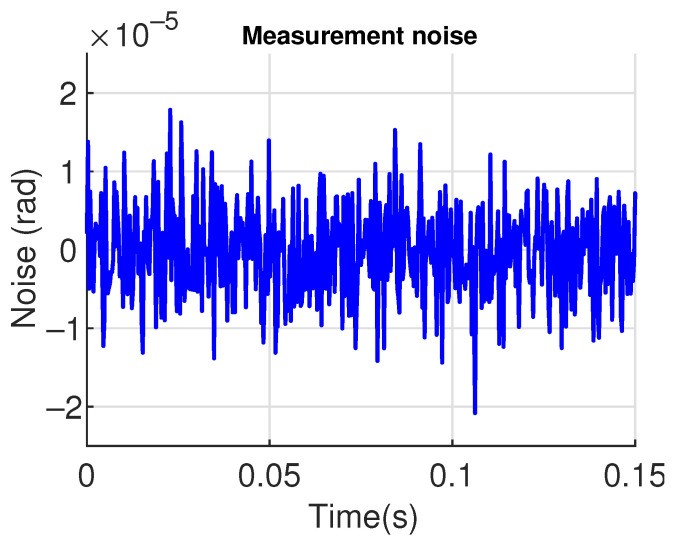
Measurement noise profile used in simulations.

For comparison purposes, the values of the control parameters for both control strategies are held the same in the two simulations, as shown in Table 2. This is for allowing the robustness analysis of the control strategies. It is important to stress that these control parameter values are applied in the three-degrees-of-freedom model of the ornithopter wing. The design parameters of Table 2 were computed based on the tuning algorithms presented in references [34,51]. As argued in [51], such parameters play an important role in the stability of the closed-loop system and then need to be tuned properly. It has been also shown in [51] that the tuning procedure can follow the well-known *root locus* method. In both simulations, a wind velocity is considered as an input disturbance (see Figure 9), and white noise is introduced as a measurement noise (Figure 10).

Since it is known that the output derivative calculation is not an appropriate measurement for practical applications (because of inevitable measurement noise), the following first-order filter is used for computing $\dot{q}$ and ${\dot{q}}_{d}$:

(77) $$H\left(s\right)=\frac{s}{\tau_{d}s+1}\;.$$

The use of a filtered derivative is imperative for the practical implementation of the PD algorithm since an exact derivative is not implementable in practical applications. However, this is not required in the current ADRC MIMO proposal, since the system states are estimated by the ESO. In addition, actuator saturation values of ±30 N·m are considered.

### 7.1. Simulation 1

**Figure 11:**
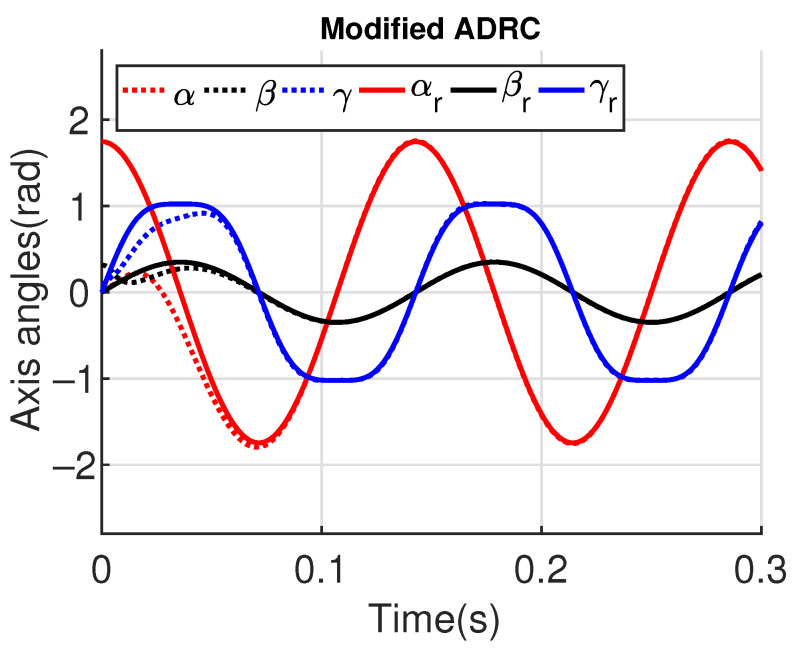
Simulation 1: Joint angles trajectories with modified ADRC.

**Figure 12:**
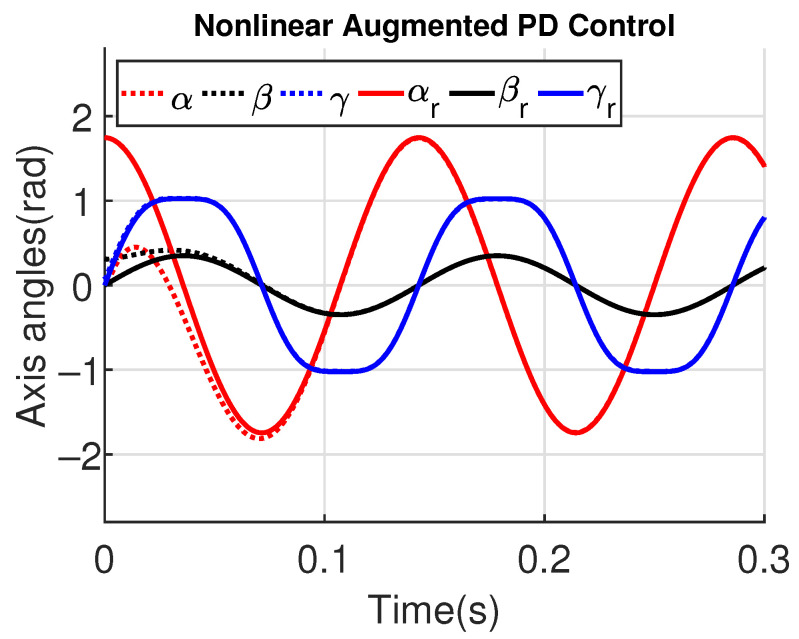
Simulation 1: Joint angles trajectories with augmented PD control ($\tau_{d}=10^{-4}$).

**Figure 13:**
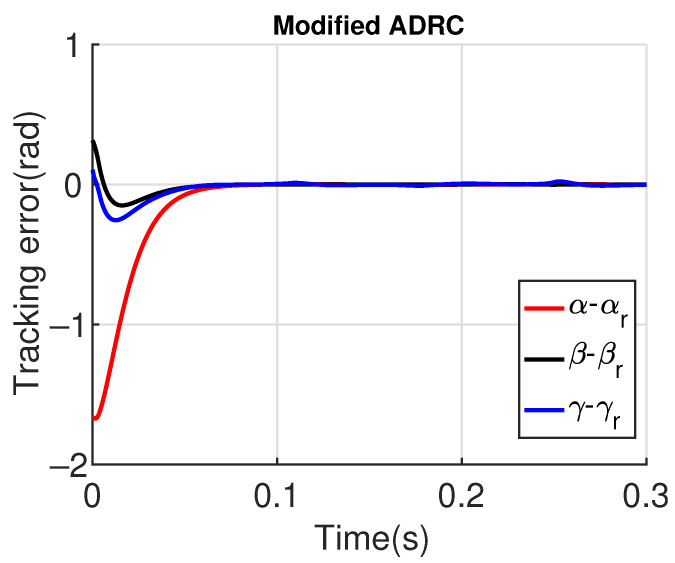
Tracking error with modified ADRC.

**Figure 14:**
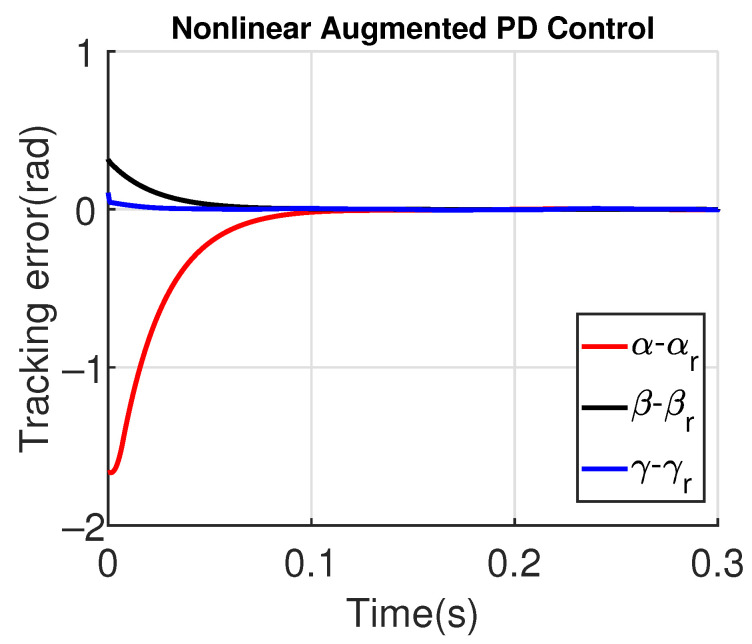
Tracking error with augmented PD control ($\tau_{d}=10^{-4}$).

**Figure 15:**
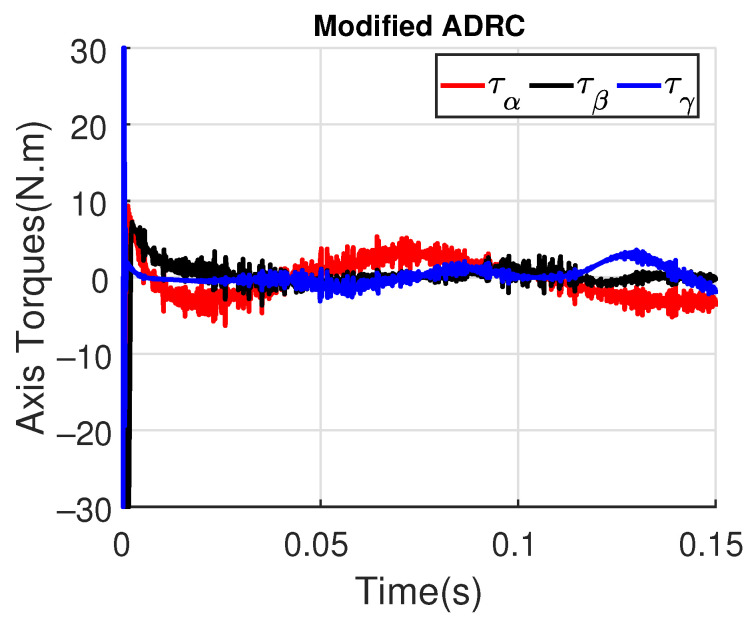
Axis Torques ($\tau_{\alpha }$, $\tau_{\beta }$, $\tau_{\gamma }$) with modified ADRC.

**Figure 16:**
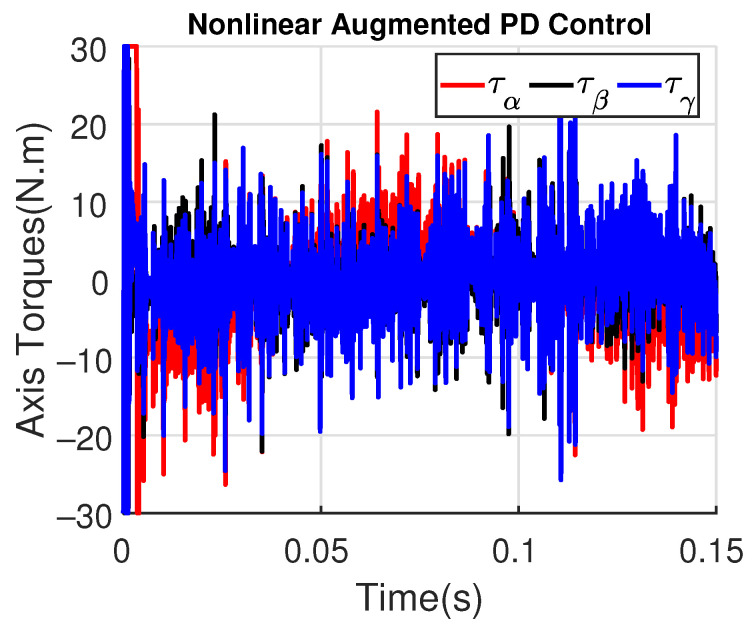
Axis Torques ($\tau_{\alpha }$, $\tau_{\beta }$, $\tau_{\gamma }$) with augmented PD control ($\tau_{d}=10^{-4}$).

Figure 11 and Figure 12 show the three wing joint angles (α, β, γ) and their reference trajectories ($\alpha_{r}$, $\beta_{r}$, $\gamma_{r}$) considering the modified ADRC and augmented PD with a time constant $\tau_{d}=10^{-4}$. The tracking errors are shown for both controllers in Figure 13 and Figure 14. Note that, by analyzing this set of four figures, the modified ADRC and augmented PD showed similar performances over time. However, as shown in Figure 15 and Figure 16, the augmented PD presents a very noisy control signal (axis torques) when compared with the modified ADRC approach. Such behavior is due to the derivative term acting as an amplifier for the measurement noise, which is a known drawback of augmented PD.

**Figure 17:**
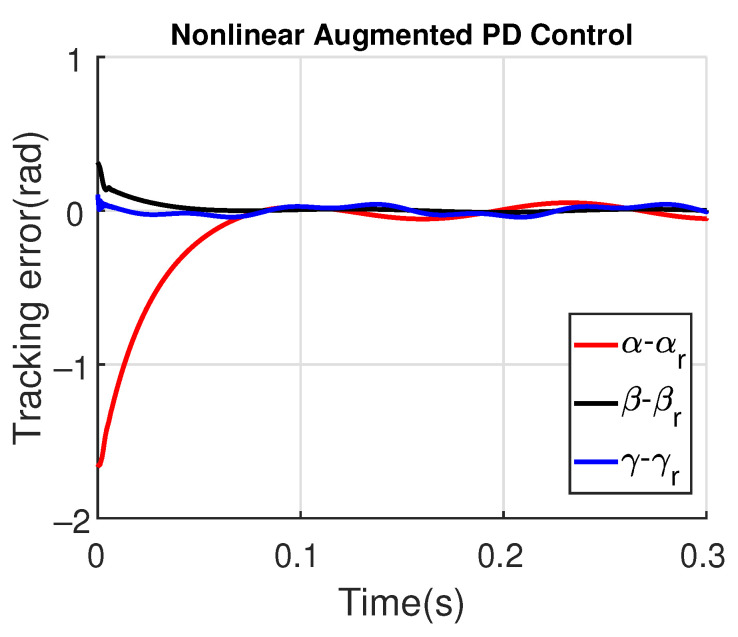
Tracking errors with augmented PD control ($\tau_{d}=10^{-3}$).

**Figure 18:**
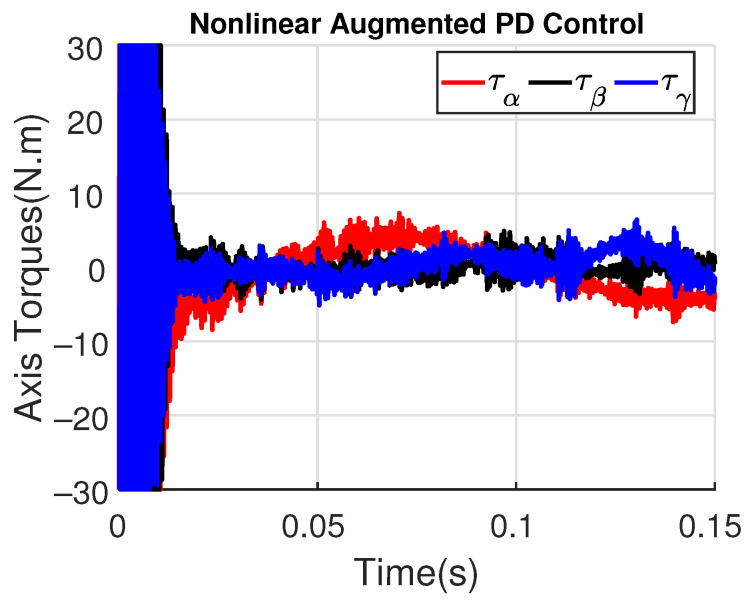
Axis Torques ($\tau_{\alpha }$, $\tau_{\beta }$, $\tau_{\gamma }$) with augmented PD control ($\tau_{d}=10^{-3}$).

For illustrating the influence of the time constant $\tau_{d}$ on the augmented PD performance, Figure 17 and Figure 18 show the tracking error and the control signal after considering $\tau_{d}=10^{-3}$.

**Figure 19:**
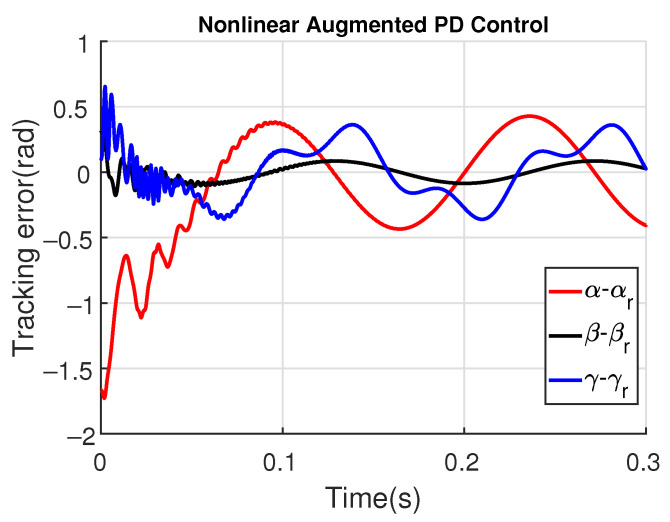
Tracking error with augmented PD control ($\tau_{d}=10^{-2}$).

**Figure 20:**
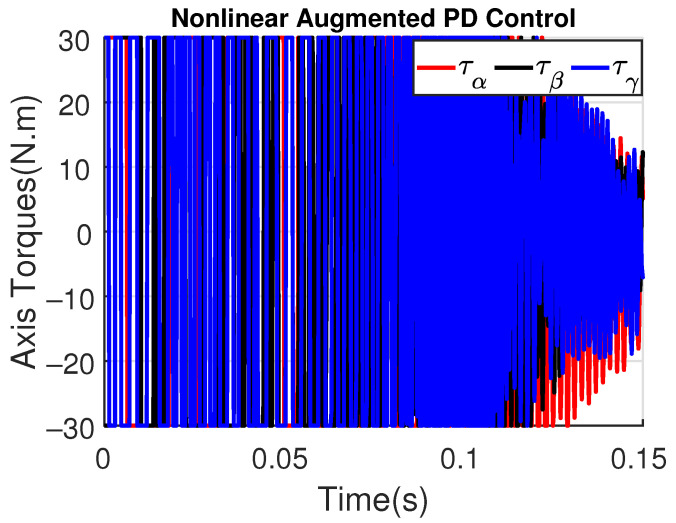
Axis Torques ($\tau_{\alpha }$, $\tau_{\beta }$, $\tau_{\gamma }$) with augmented PD control ($\tau_{d}=10^{-2}$).

Note that the tracking error amplitude is greater than those shown in Figure 13 and Figure 14. Moreover, although the control signal has a minor noise amplitude, its performance is worst in the initial instants, also presenting a switching behavior due to the saturation levels of ±30 N.m. As shown in Figure 19 and Figure 20, the augmented PD performance is degraded even more when $\tau_{d}=10^{-2}$, which is expected due to the worse approximation of the derivative term.

### 7.2. Simulation 2

**Figure 21:**
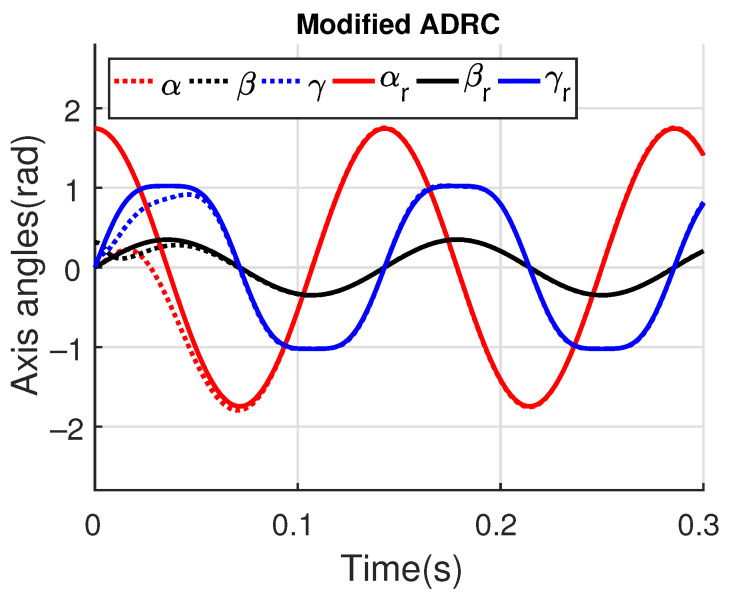
Simulation 2: Joint angles trajectories with modified ADRC.

**Figure 22:**
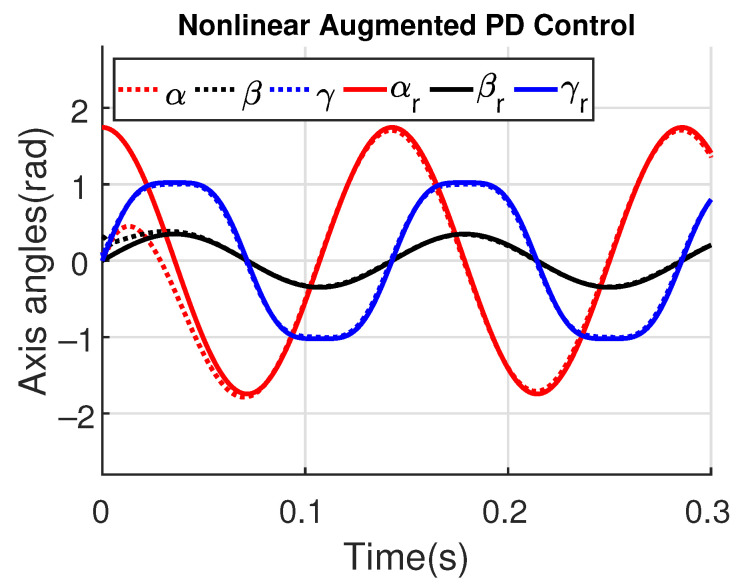
Simulation 2: Joint angles trajectories with augmented PD control ($\tau_{d}=10^{-3}$).

**Figure 23:**
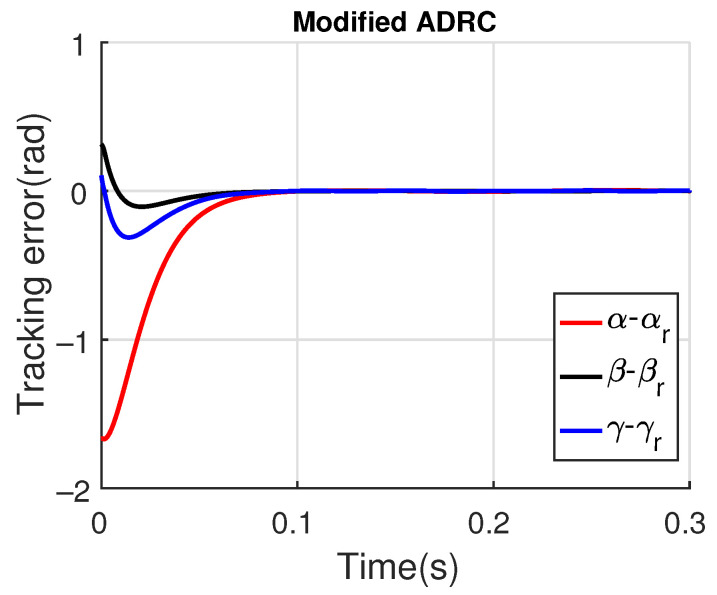
Simulation 2: Tracking error with modified ADRC.

**Figure 24:**
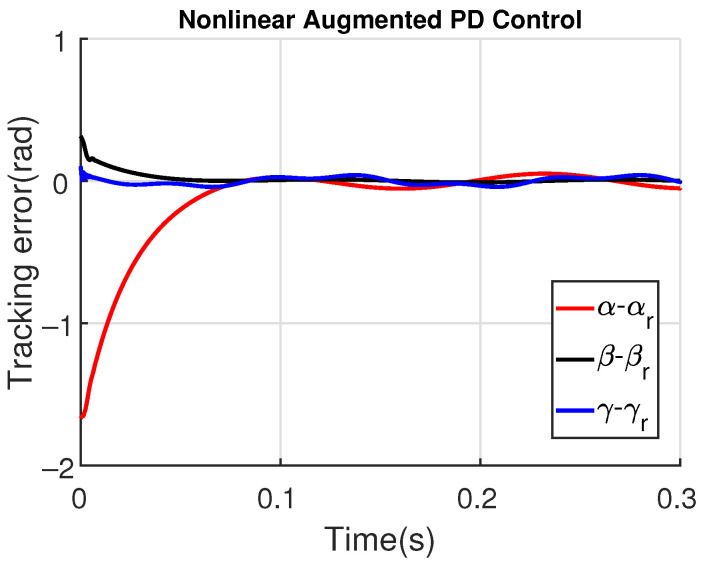
Simulation 2: Tracking errors with augmented PD control ($\tau_{d}=10^{-3}$).

**Figure 25:**
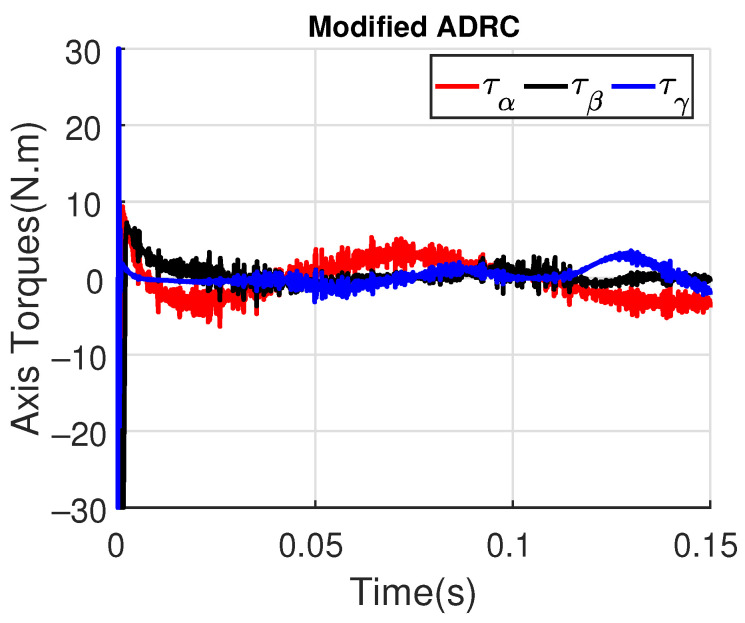
Simulation 2: Axis Torques ($\tau_{\alpha }$, $\tau_{\beta }$, $\tau_{\gamma }$) with modified ADRC.

**Figure 26:**
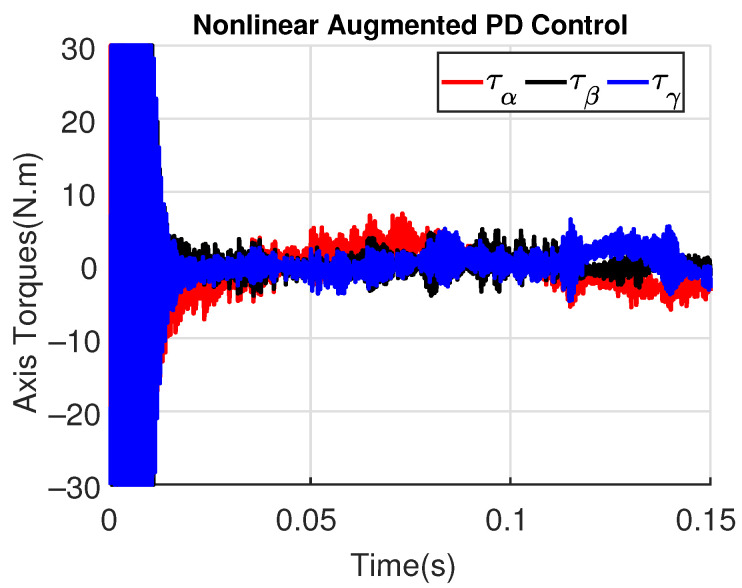
Simulation 2: Axis Torques ($\tau_{\alpha }$, $\tau_{\beta }$, $\tau_{\gamma }$) with augmented PD control ($\tau_{d}=10^{-3}$).

Figure 21, Figure 22, Figure 23, Figure 24, Figure 25 and Figure 26 show the simulation results when some wing parameters are changed (see Table 1). Note that both strategies showed good robustness to the variation of dynamical parameters. However, as in simulation 1, the modified ADRC again presented a smaller tracking error amplitude and a resulting control signal with less noise.

Therefore, the simulation results show that the modified ADRC presented a similar tracking error performance as the augmented PD with $\tau_{d}=10^{-4}$, but the augmented PD control signal has minor robustness to measurement noise, presenting a significantly noisier control signal. By using $\tau_{d}=10^{-3}$, the augmented PD control signal noise is reduced, although it was also higher than that of the modified ADRC. However, the tracking error performance becomes more significant in terms of small amplitude.

Thus, besides its simplicity, since the augmented PD strategy needs a complex mathematical description of the controlled system, the modified ADRC presented a better performance, mainly due to its better robustness to measurement noise, presenting a less noisy control signal.

## 8. Conclusions

This paper discusses a design procedure for the control of MIMO uncertain systems as an extension of the modified ADRC strategy for SISO plants. The proposed strategy, which, in the SISO case, is known for its robustness properties against parametric uncertainties, unmodeled dynamics, and external disturbances, also revealed good performance for MIMO plants with coupled input variables. Developments show that the only information required is the sign of the main diagonal entries of the control matrix. The relevance of the MP-ADRC method compared to LADRC lies in the fact that the former does not depend on the control gain value *B* of the plant. Even in the LADRC strategy, decoupled control gains are required both for the ESO design and for the law of formation of the control variable. In the MIMO MP-ADRC, although the approach considers a matrix gain *B*, this is not required either in the ESO project or in the definition of the control law, as can be verified by Equations (34) and (43). In order to illustrate the efficiency of the proposed strategy, the methodology was applied to the wing attitude control problem of an ornithopter. Then, simulation trials were performed and compared with the extended PD scheme. After analyzing the obtained results, it was observed that both controllers’ performance was similar regarding tracking error and robustness to uncertain parameters. However, the extended PD presented a noisier control signal due to the derivative effect. The use of filtered derivatives is imperative for the implementation of the PD algorithm. Moreover, unlike the ADRC, the robustness of the PD control proved to be dependent on $\tau_{d}$ in the Equation (77), as can be observed from Figure 19 and Figure 20, where the performance has deteriorated as $\tau_{d}$ increased. On the other hand, the control signal is noisier for small $\tau_{d}$, as can be observed from Figure 16, which is very critical in practical applications. Therefore, the simulation results showed that the MP-ADRC has the advantage of good robustness with a less noisy control signal when compared with augmented PD control. Then, in addition to being simpler to implement, it is possible to conclude, from the simulation results, that the MP-ADRC MIMO showed a better general performance after analyzing the tracking error and the control signal.

## Figures and Tables

**Table 1:** Parameters of the ornithopter wing model.

	Wing Span ($L_{a}$)	Ornithopter Mass ($M_{p}$)	Wing Mass $M_{a}$	Wing Chord ($C_{a}$)
Simulation 1	0.279 m	0.316 kg	0.0274 kg	0.112 m
Simulation 2	0.140 m	0.858 kg	0.0823 kg	0.056 m

**Table 2:** Controller parameters used in simulations.

MP-ADRC			Computed Torque (Augmented PD)	
Filter poles, *p*	ESO poles, $\omega_{o}$	Output gain, $\beta_{o}$	$\;K_{p}\;$	$K_{v}$
80	$3\times 10^{4}$	5000	4096	89.6

## Data Availability

Data sharing is not applicable to this article.

## Abbreviations

The following abbreviations are used in this manuscript:

ADRC	Active Disturbance Rejection Control
BIBO	Bounded Input Bounded Output
ESO	Extended State Observer
LHP	Left Half-Plane (Complex)
LQI	Linear Quadratic Integrator
LQR	Linear Quadratic Regulator
MIMO	Multiple-Input Multiple-Output
MP-ADRC	Modified-Plant Active Disturbance Rejection Control
MPC	Model Predictive Control
PD	Proportional-Derivative
PID	Proportional-Integral-Derivative
SISO	Single-Input Single-Output
SMC	Sliding-Mode Control
VS-MRAC	Variable Structure Model Reference Adaptive Control

## Appendix A. System’s Dynamical Parameters

In the following, we highlight the variable elements of the model in Equation (1). For simplifying notations, in the sequence we adopt the reduced forms for the trigonometric functions, namely:

$$cq_{i}=\cos\left(q_{i}\left(t\right)\right),\;cq_{i}^{2}=\cos{\left(q_{i}\left(t\right)\right)}^{2},\;sq_{i}=\sin\left(q_{i}\left(t\right)\right)\;\mathrm{and}\;sq_{i}^{2}=\sin{\left(q_{i}\left(t\right)\right)}^{2}.$$

- $q^{T}=\left[\alpha ,\beta ,\gamma \right]$ are the joints’ rotation angles around the *Z*, *Y*, and *X* axes, respectively (Euler angles);
- $\tau^{T}=\left[\tau_{\alpha },\tau_{\beta },\tau_{\gamma }\right]$ is the vector of actuator torques at the three wing joints;
- $M\left(q,\dot{q}\right)\in I\;R^{3\times 3}$ is a symmetric positive definite inertia matrix with entries $m_{ij}$ (*i* rows and *j* columns) described by
  $$\begin{array}{cc} m_{11} & =\frac{m\;\left(C^{2}+3\;l^{2}\;{cq_{2}}^{2}+4\;l^{2}\right)}{12}\;,\;\;m_{12}=0\;,\;\;m_{13}=\frac{m\;sq_{2}\;\left(C^{2}+4\;l^{2}\right)}{12}\;,\;\;m_{21}=0\\ m_{22} & =\frac{m\;\left(-C^{2}\;{cq_{1}}^{2}+C^{2}+4\;l^{2}\;{cq_{1}}^{2}+3\;l^{2}\right)}{12}\;,\;\;m_{23}=\frac{m\;cq_{1}\;cq_{2}\;sq_{1}\;\left(C^{2}-4\;l^{2}\right)}{12}\;,\\ m_{31} & =\frac{m\;sq_{2}\;\left(C^{2}+4\;l^{2}\right)}{12}\;,\;\;m_{32}=\frac{m\;cq_{1}\;cq_{2}\;sq_{1}\;\left(C^{2}-4\;l^{2}\right)}{12}\;,\\ m_{33} & =\frac{m\;\left(C^{2}\;{cq_{1}}^{2}\;{cq_{2}}^{2}+C^{2}\;{sq_{2}}^{2}+4\;l^{2}\;{cq_{2}}^{2}\;{sq_{1}}^{2}+4\;l^{2}\;{sq_{2}}^{2}\right)}{12}\;; \end{array}$$
- $C_{q}\left(q,\dot{q}\right)\in I\;R^{3\times 3}$ is the Coriolis matrix with entries
  $$\begin{array}{cc} c_{11} & =0\;,\;\;c_{12}=\frac{{\dot{q}}_{3}\;m\;cq_{2}\;\left(-C^{2}\;{cq_{1}}^{2}+C^{2}+4\;l^{2}\;{cq_{1}}^{2}\right)}{6}-\frac{{\dot{q}}_{2}\;m\;s2q_{1}\;\left(C^{2}-4\;l^{2}\right)}{24}\\ & -\frac{{\dot{q}}_{1}\;l^{2}\;m\;\sin\left(2\;q_{2}\right)}{4}\;,c_{13}=\frac{{\dot{q}}_{3}\;m\;cq_{1}\;{cq_{2}}^{2}\;sq_{1}\;\left(C^{2}-4\;l^{2}\right)}{12}\;,\\ c_{21} & =\frac{{\dot{q}}_{1}\;l^{2}\;m\;\sin\left(2\;q_{2}\right)}{8}-\frac{{\dot{q}}_{3}\;m\;cq_{2}\;\left(-C^{2}\;{cq_{1}}^{2}+C^{2}+4\;l^{2}\;{cq_{1}}^{2}\right)}{6}+\frac{{\dot{q}}_{2}\;m\;s2q_{1}\;\left(C^{2}-4\;l^{2}\right)}{12}\;,\\ c_{22} & =0\;,\;\;c_{23}=-\frac{{\dot{q}}_{3}\;m\;\sin\left(2\;q_{2}\right)\;\left(2\;l^{2}\;c2q_{1}-\frac{C^{2}\;c2q_{1}}{2}+\frac{C^{2}}{2}+2\;l^{2}\right)}{24}\;,\;\;\\ c_{31} & =-\frac{{\dot{q}}_{3}\;m\;cq_{1}\;{cq_{2}}^{2}\;sq_{1}\;\left(C^{2}-4\;l^{2}\right)}{6}\;,\\ c_{32} & =\frac{{\dot{q}}_{1}\;m\;cq_{2}\;\left(C^{2}\;{cq_{1}}^{2}-4\;l^{2}\;{cq_{1}}^{2}+4\;l^{2}\right)}{6}-\frac{{\dot{q}}_{2}\;m\;cq_{1}\;sq_{1}\;sq_{2}\;\left(C^{2}-4\;l^{2}\right)}{12}\\ \; & +\frac{{\dot{q}}_{3}\;m\;cq_{2}\;sq_{2}\;\left(2\;l^{2}\;\left(2\;{cq_{1}}^{2}-1\right)+\frac{C^{2}}{2}+2\;l^{2}-\frac{C^{2}\;\left(2\;{cq_{1}}^{2}-1\right)}{2}\right)}{6}\;,\;\;c_{33}=0\;; \end{array}$$
- $N{\left(q\right)}^{T}=\left[\begin{array}{ccc} 0 & 49\;l\;m\;cq_{2}/10 & 0 \end{array}\right]$ includes gravity terms and other forces which act at the joints;
- $\tau_{v}^{T}=\left[\tau_{v1},\tau_{v2},\tau_{v3}\right]$ is the vector of aerodynamic forces, being
  $$\begin{array}{cc} \tau_{v1} & =\frac{{Ø}_{\mathrm{vb}}\;\left(\frac{70,479\;l\;{Ø}_{\mathrm{va}}\;cq_{3}\;C^{3}}{80,000}+\frac{123\;l\;{Ø}_{\mathrm{va}}\;C^{2}}{5000}\right)}{\sqrt{{Ø}_{\mathrm{va}}}}\;,\;\tau_{v2}=-\frac{70,479\;C^{2}\;l^{2}\;\sqrt{{Ø}_{\mathrm{va}}}\;{Ø}_{\mathrm{vb}}\;cq_{3}}{20,000}\;,\;\\ \tau_{v3} & =-\frac{70,479\;C^{2}\;l^{2}\;\sqrt{{Ø}_{\mathrm{va}}}\;{Ø}_{\mathrm{vb}}\;sq_{3}}{20,000}\;,\\ \tau_{va} & ={\left(cq_{2}\;l\;{\dot{q}}_{2}-\tau_{va4}\right)}^{2}+{\left(\tau_{va1}\;\left(cq_{1}\;{\dot{q}}_{2}-cq_{2}\;sq_{1}\;{\dot{q}}_{3}\right)-{Ø}_{\mathrm{va}2}\;\left(\tau_{va5}-cq_{2}\;l\;sq_{1}\right)\right)}^{2}+{\tau_{va3}}^{2}\;,\\ \tau_{vb} & =sq_{2}\;\tau_{vb3}+cq_{1}\;cq_{2}\;\left(V_{ix}+\tau_{vb2}\;\left(\tau_{vb4}-cq_{2}\;l\;sq_{1}\right)-\tau_{vb1}\;\left(cq_{1}\;{\dot{q}}_{2}-cq_{2}\;{\dot{q}}_{3}\;sq_{1}\right)\right)+\\ \; & cq_{2}\;sq_{1}\;\left(V_{ix}+\tau_{vb2}\;\left(\tau_{vb5}+cq_{1}\;cq_{2}\;l\right)-\tau_{vb1}\;\left({\dot{q}}_{2}\;sq_{1}+cq_{1}\;cq_{2}\;{\dot{q}}_{3}\right)\right)\;,\\ \tau_{va1} & =l\;sq_{2}-\frac{C\;cq_{2}\;cq_{3}}{4}\;,\;\;\tau_{va2}=v_{1}\left(t\right)+sq_{2}\;{\dot{q}}_{3}\;,\\ \tau_{va3} & =V_{iy}-\tau_{va1}\;\left(sq_{1}\;{\dot{q}}_{2}+cq_{1}\;cq_{2}\;{\dot{q}}_{3}\right)+\tau_{va2}\;\left(\tau_{va6}+cq_{1}\;cq_{2}\;l\right)\;,\\ \tau_{va4} & =\frac{C\;cq_{2}\;cq_{3}\;{\dot{q}}_{3}}{4}+\frac{C\;sq_{2}\;sq_{3}\;{\dot{q}}_{2}}{4}\;,\;\tau_{va5}=\frac{C\;\left(cq_{1}\;cq_{3}-sq_{1}\;sq_{2}\;sq_{3}\right)}{4}\;, \end{array}$$
  $$\begin{array}{cc} \tau_{va6} & =\frac{C\;\left(cq_{3}\;sq_{1}+cq_{1}\;sq_{2}\;sq_{3}\right)}{4}\;,\\ \tau_{vb1} & =l\;sq_{2}-\frac{C\;cq2\;sq_{3}}{4}\;,\;\;\tau_{vb2}={\dot{q}}_{1}+sq_{2}\;{\dot{q}}_{3}\;,\;\;\\ \tau_{vb3} & =\left(V_{ix}+cq_{2}\;{\dot{q}}_{2}\;l-\frac{C\;cq_{2}\;cq_{3}\;{\dot{q}}_{3}}{4}+\frac{C\;{\dot{q}}_{2}\;sq_{2}\;sq_{3}}{4}\right)\;,\\ \tau_{vb4} & =\frac{C\;\left(cq_{1}\;cq_{3}-sq_{1}\;sq_{2}\;sq_{3}\right)}{4}\;,\;\;\tau_{vb5}=\frac{C\;\left(cq_{3}\;sq_{1}+cq_{1}\;sq_{2}\;sq_{3}\right)}{4}\;; \end{array}$$
- $V_{i}^{T}=\left[V_{ix}\;,V_{iy}\;,V_{iz}\right]$ is the wind velocity vector with components $V_{ix}$, $V_{iy}$, $V_{iz}$ on the xyz axes.
